# Supplementary figures and images for: Induction of apoptosis by double-stranded RNA was present in the last common ancestor of cnidarian and bilaterian animals
Source: PLoS Pathog. 2024 Jul 16;20(7):e1012320. doi: 10.1371/journal.ppat.1012320 (PMC11251625; doi:10.1371/journal.ppat.1012320)

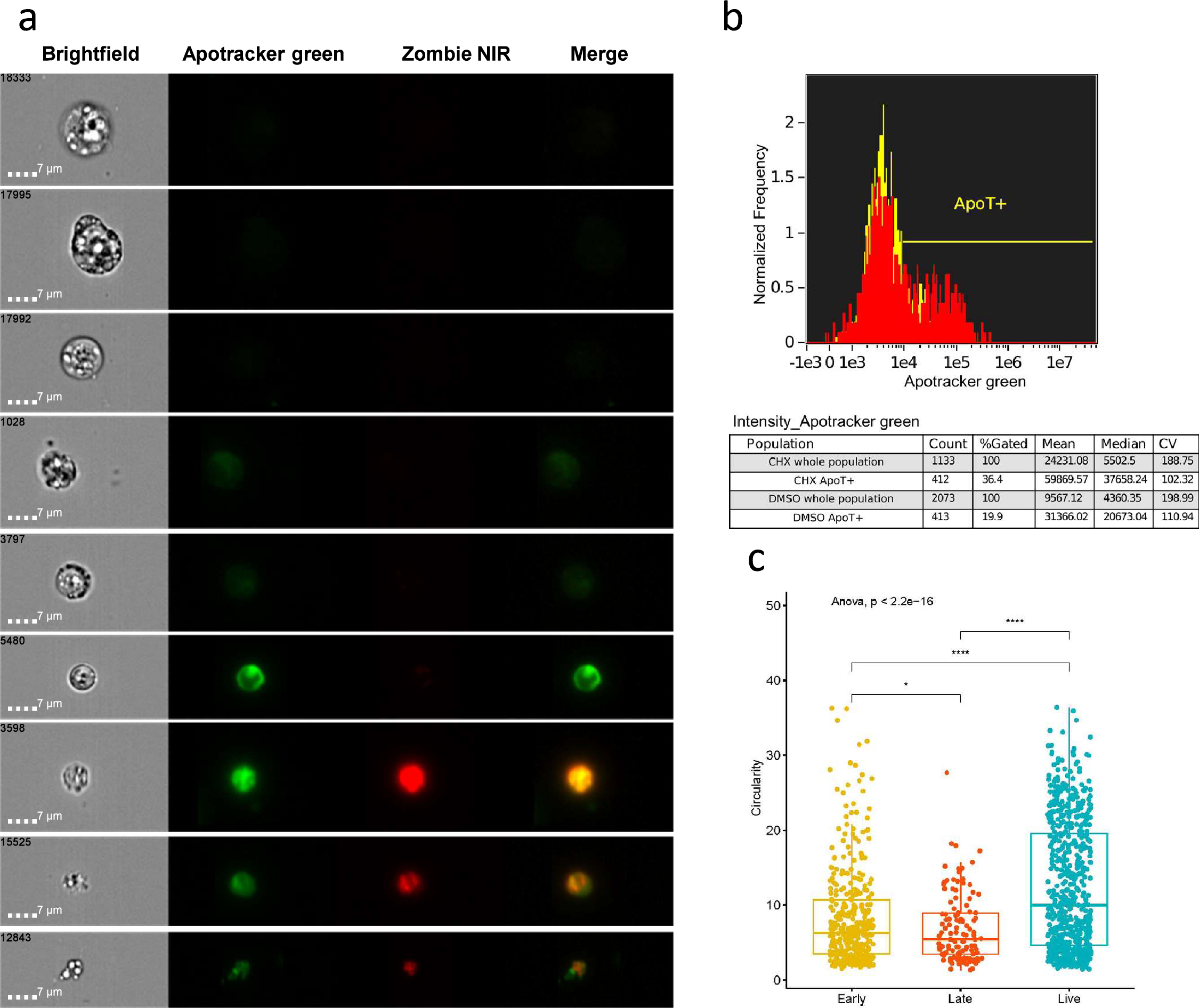

Supplement: S1 Fig — N. vectensis zygotes were treated with 2 mM cycloheximide (CHX) or an equivalent concentration of DMSO for 48 hours. Cells were dissociated and stained with Apotracker Green and the viability dye Zombie NIR. (A) Representative images of cells that were negative to Apotracker Green and Zombie NIR (first 3 rows), cells that were positive to Apotracker Green (rows 4–6), and cells that were positive to both Apotracker Green and Zombie NIR (last 3 rows). (B) Histogram comparing the difference in Apotracker Green intensity in DMSO versus CHX treated cells. The summary statistics is shown in the table. (C) Comparison of circularity score across cells that are negative to both markers (live), positive to Apotracker Green (early apoptosis), and positive to both markers (late apoptosis). One way ANOVA test with Tukey’s post hoc test was performed. Individual data points (single cells) are shown as a jitter. * p<0.05, **p<0.01, ***p<0.001. (TIF) [file ppat.1012320.s001.tif]

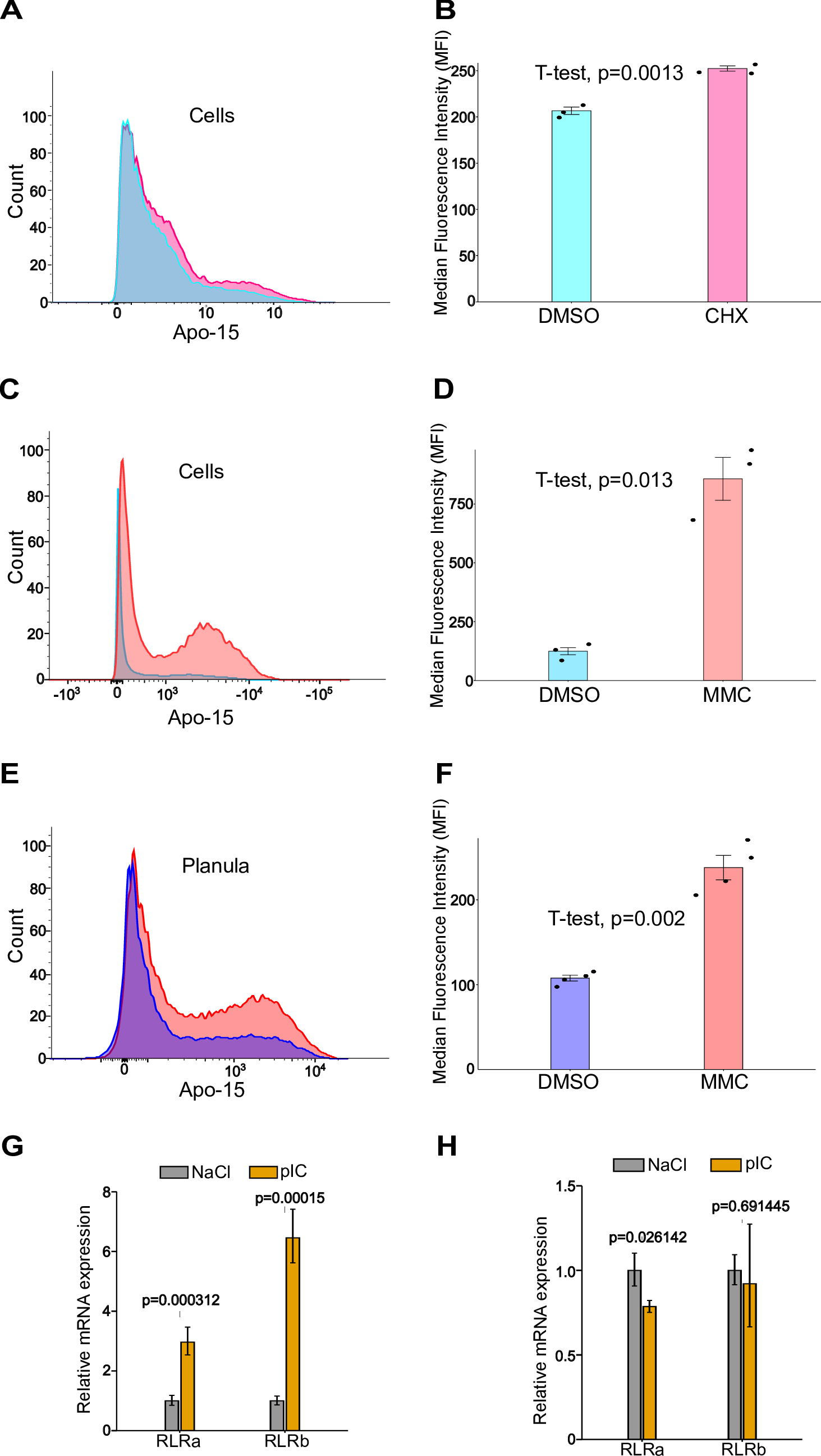

Supplement: S2 Fig — (A) Cells were dissociated from 48 hours old planulae and treated with 2mM cycloheximide or DMSO for 48 hours. Cells were than stained with Apotracker Green and subjected to flow cytometry analysis. Representative result is shown. (B) quantification of (A). Three biological replicates were used. Error bars indicate standard deviation. Cells were dissociated from 48 hours old planulae and treated with 60uM MMC or DMSO control. (C) Representative histogram showing the difference between DMSO and MMC treated cells. (D) Median fluorescence intensity (MFI) in DMSO versus MMC treated cells. (E) Zygotes were incubated with mitomycin-c or DMSO for 48 hours. Cells were then dissociated and subjected to flow cytometry analysis. Representative result is shown. (F) quantification of (E). Four biological replicates were used. (G) Zygotes were injected with poly(I:C) and the mRNA expression levels of RLRa and RLRb were measured by RT-qPCR. (H) Zygotes were incubated with a high concentration of poly(I:C) (0.5 ug/uL) and subjected to RT-qPCR as described in (G). Error bars indicate standard deviation. All comparisons were done by two-sided t-test. Individual data points are shown as a jitter. * p<0.05, **p<0.01, ***p<0.001. (TIF) [file ppat.1012320.s002.tif]

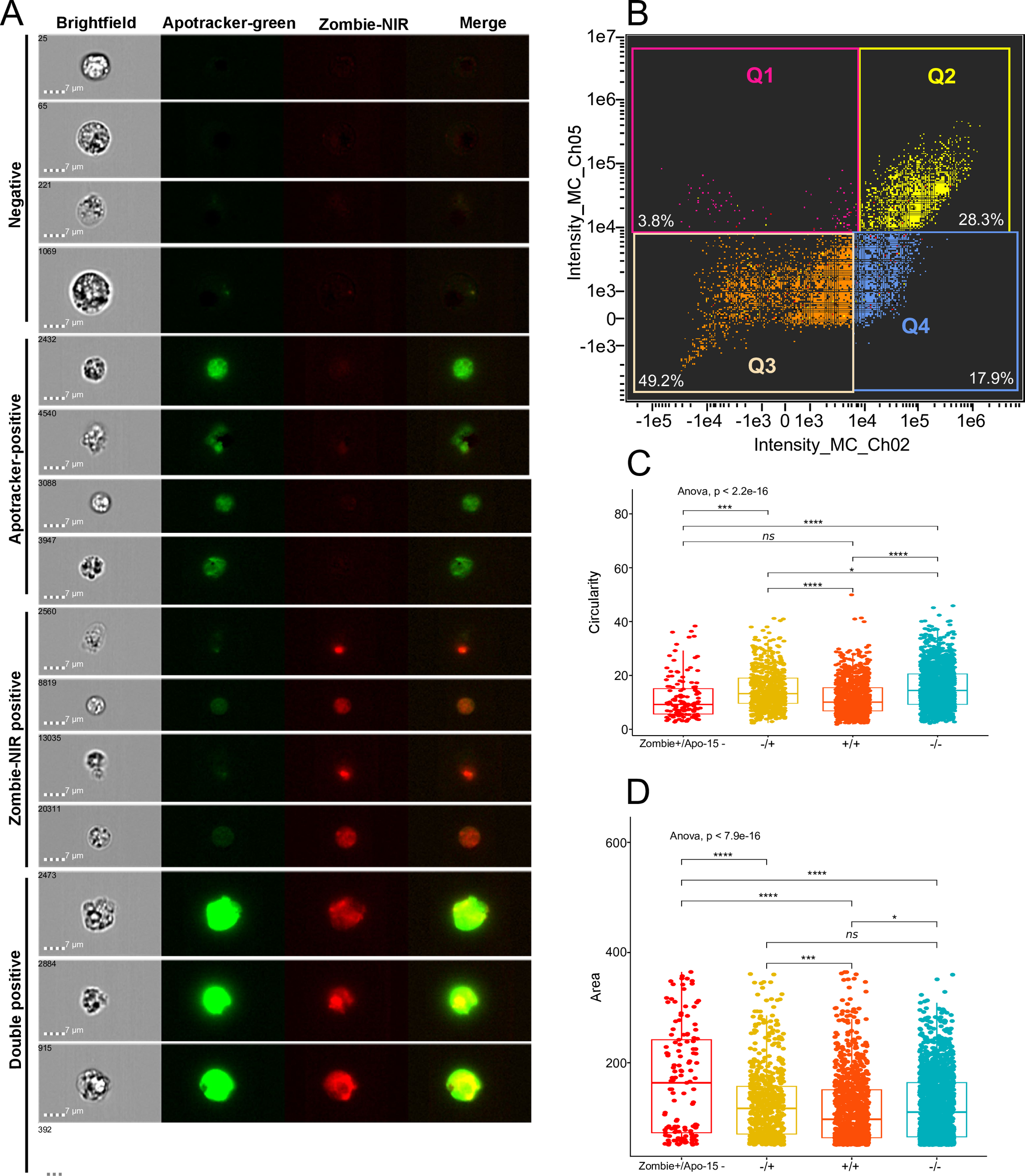

Supplement: S3 Fig — Cells were dissociated and stained with Apotracker Green and the viability dye Zombie NIR. (A) Representative images of cells that were negative to both Apotracker Green and Zombie NIR (first 4 rows), cells that were positive to Apotracker Green and negative to zombie NIR (rows 5–8), cells that were positive to Zombie NIR and negative to Apotracker Green (rows 9–12), and cells that were positive to both Apotracker Green and Zombie NIR (rows 13–16). (B) Cells were gated based on their signal to Apotracker Green (X axis) and Zombie NIR (Y axis). Percentage for each quadrant is shown. Q1: Apotracker Green negative/ Zombie NIR positive, Q2: Apotracker Green positive/ Zombie NIR positive, Q3: Apotracker Green negative/ Zombie NIR negative, Q4: Apotracker Green positive/ Zombie NIR negative (C) Comparison of circularity scores across cells that are (left to right): positive to Zombie NIR only (dead/necrotic) (Q1), positive to Apotracker Green only (early apoptosis) (Q4), positive for both markers (late apoptosis/ and/or necrosis) (Q2), negative to both markers (Q3) (live). (D) Area of cells belonging to each population as described in (C). One way ANOVA test with Tukey’s post hoc test was performed. Individual data points (single cells) are shown as a jitter. * p<0.05, **p<0.01, ***p<0.001. (TIF) [file ppat.1012320.s003.tif]

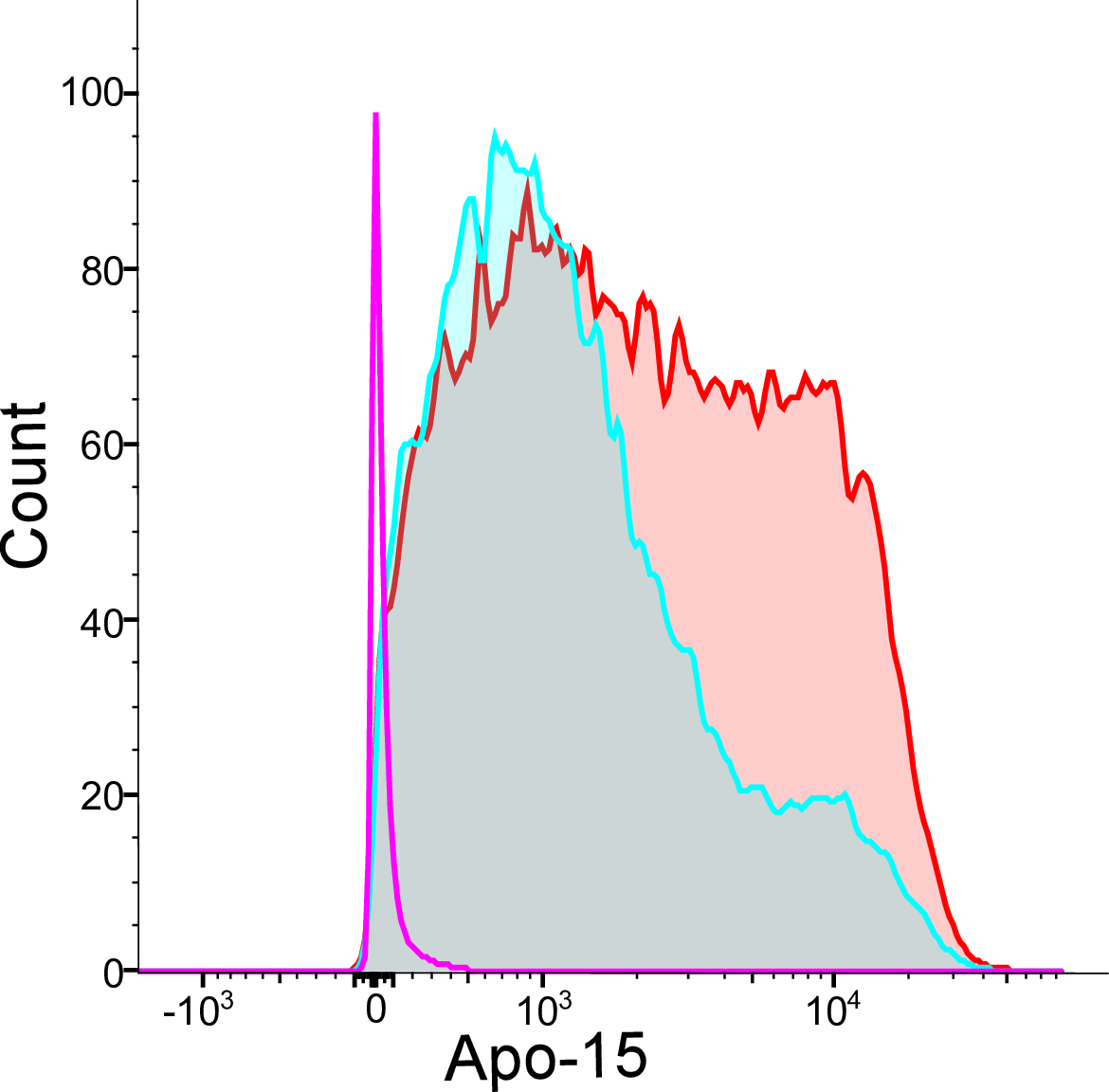

Supplement: S4 Fig — (A) a histogram showing the green fluorescent signal of SYTOX Blue single stained mock transfected cells (purple line), mock transfected cells stained with both SYTOX Blue and Apotracker Green (cyan), and poly(I:C) transfected cells stained with both SYTOX Blue and Apotracker Green (red). (TIF) [file ppat.1012320.s004.tif]

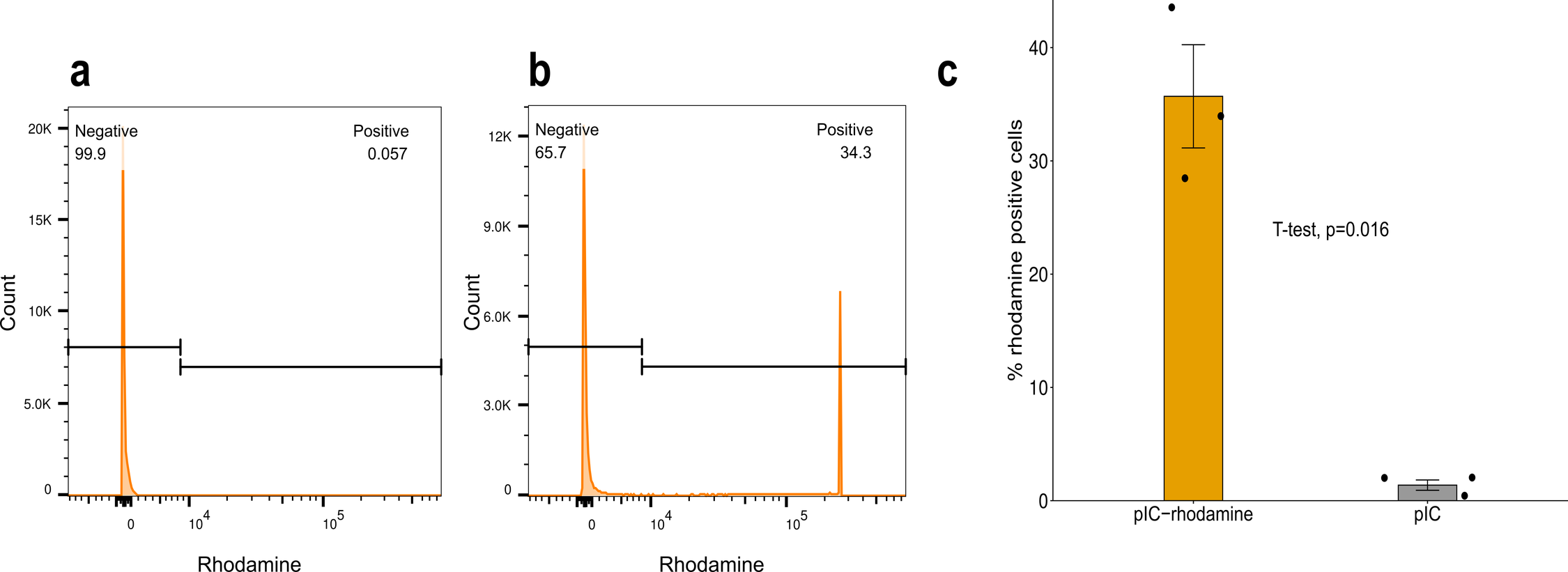

Supplement: S5 Fig — Dissociated cells were seeded overnight and transfected using lipofectamine 3000. Transfection efficiency was determined after 24 hours by flow cytometry analysis. (A) representative result of cells transfected with 2 μg/ml unlabeled poly(I:C). (B) representative result of cells transfected with 2 μg/ml rhodamine labeled poly(I:C). (C) Quantification of (A) and (B). Error bars indicate standard deviation. 30,000 events were acquired for each replicate. 3 biological replicates were performed for each condition. (TIF) [file ppat.1012320.s005.tif]

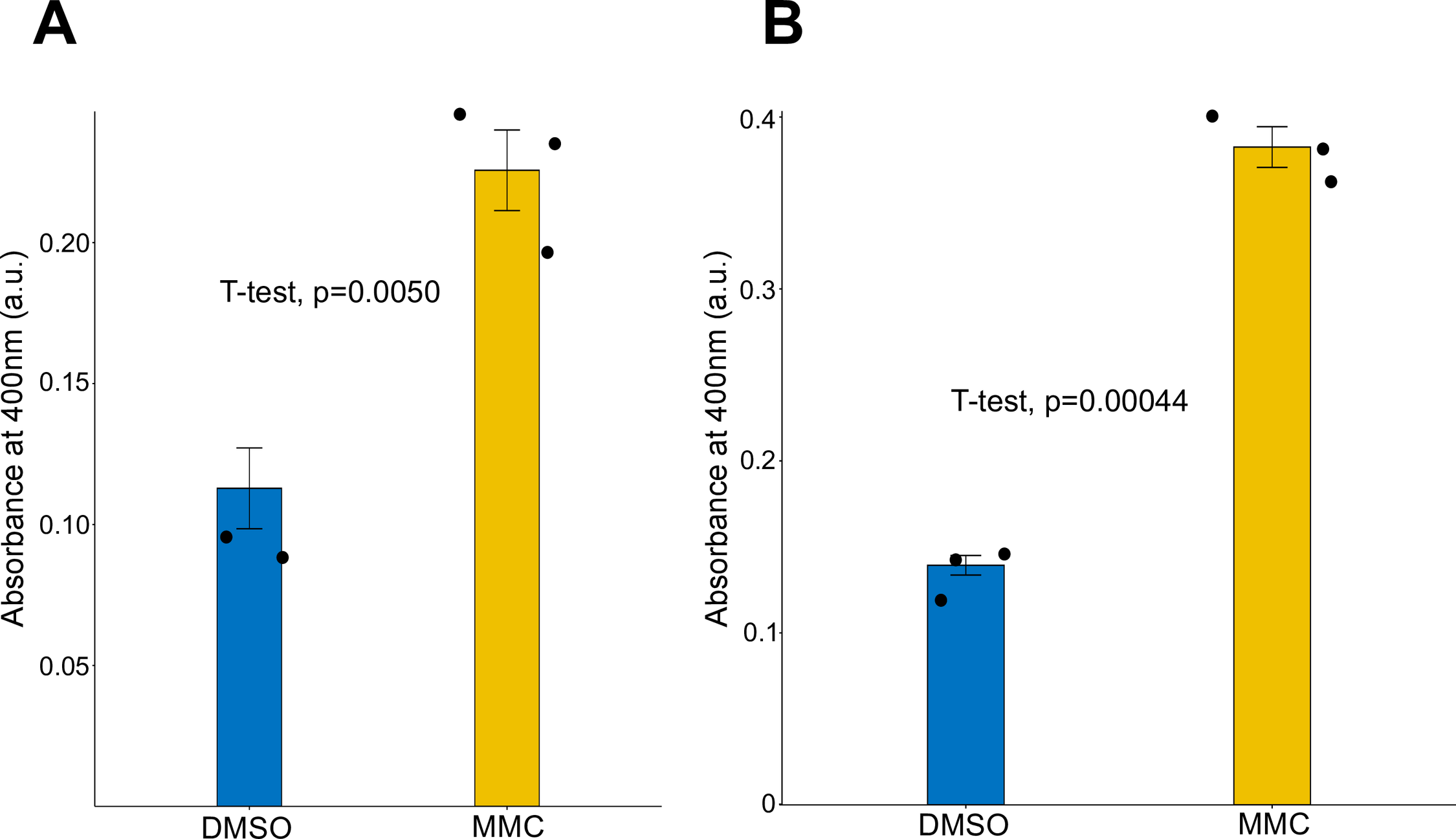

Supplement: S6 Fig — Optical density was measured at 400nm and is shown in arbitrary units. (A) Lysates were obtained from DMSO and their MMC treated counterparts at 24 hours, and (B) at 48 hours after treatment. The graphs represent the average of 3 biological replicates. Two-sided t test was performed. Error bars represent standard deviation. Individual data points are shown as a jitter. * p<0.05, **p<0.01, ***p<0.001. (TIF) [file ppat.1012320.s006.tif]

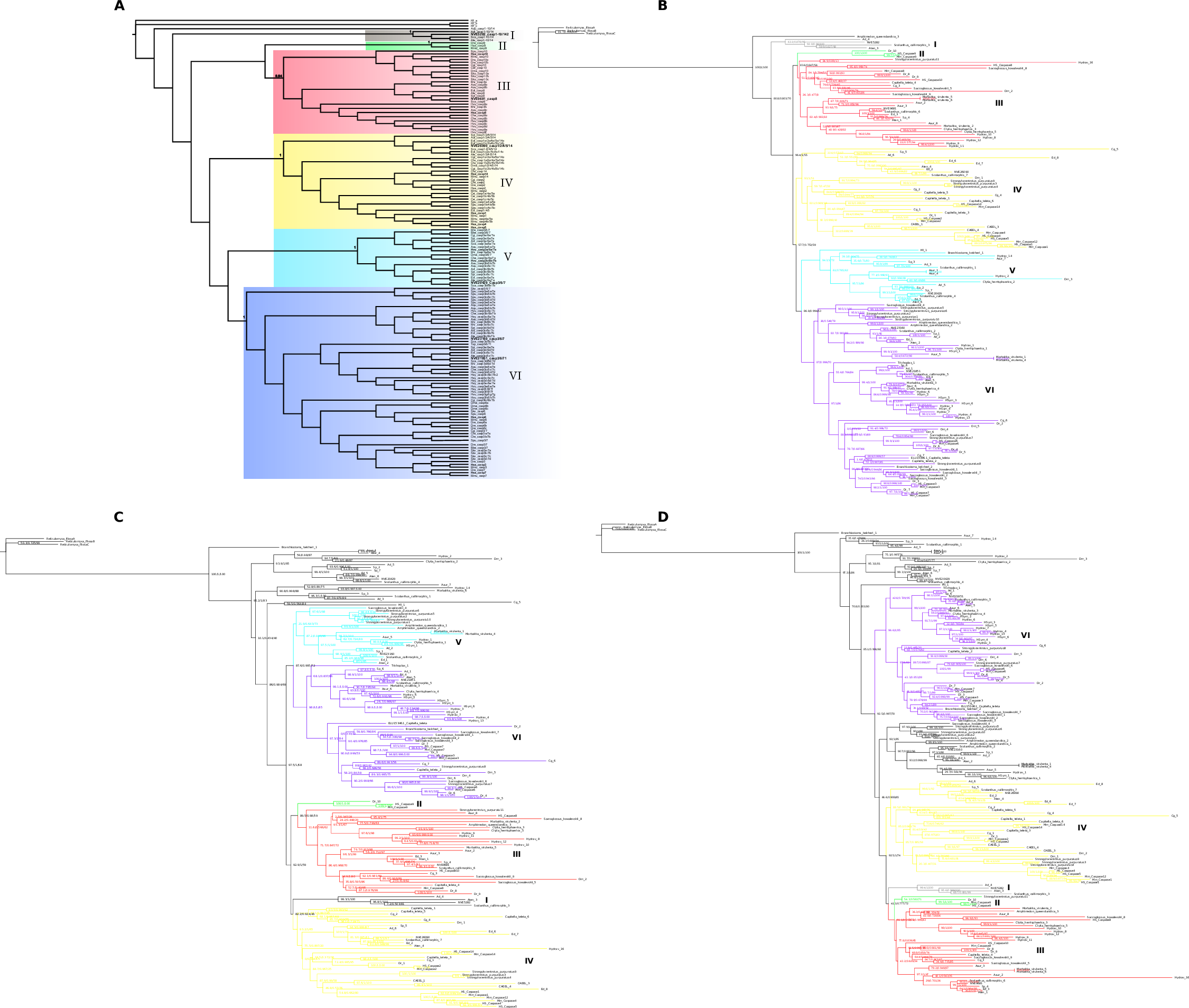

Supplement: S7 Fig — (A) Topology generated through Bayesian analysis of the full-length sequence alignment for the caspase protein by MAFFT. (B) Topology from maximum likelihood analysis using a MAFFT alignment of the caspase domain (Casc). (C) Topology obtained by maximum likelihood using a muscle alignment of the full-length caspase sequence. (D) Topology obtained by maximum likelihood using a muscle alignment of the caspase domain (Casc). (TIF) [file ppat.1012320.s007.tif]

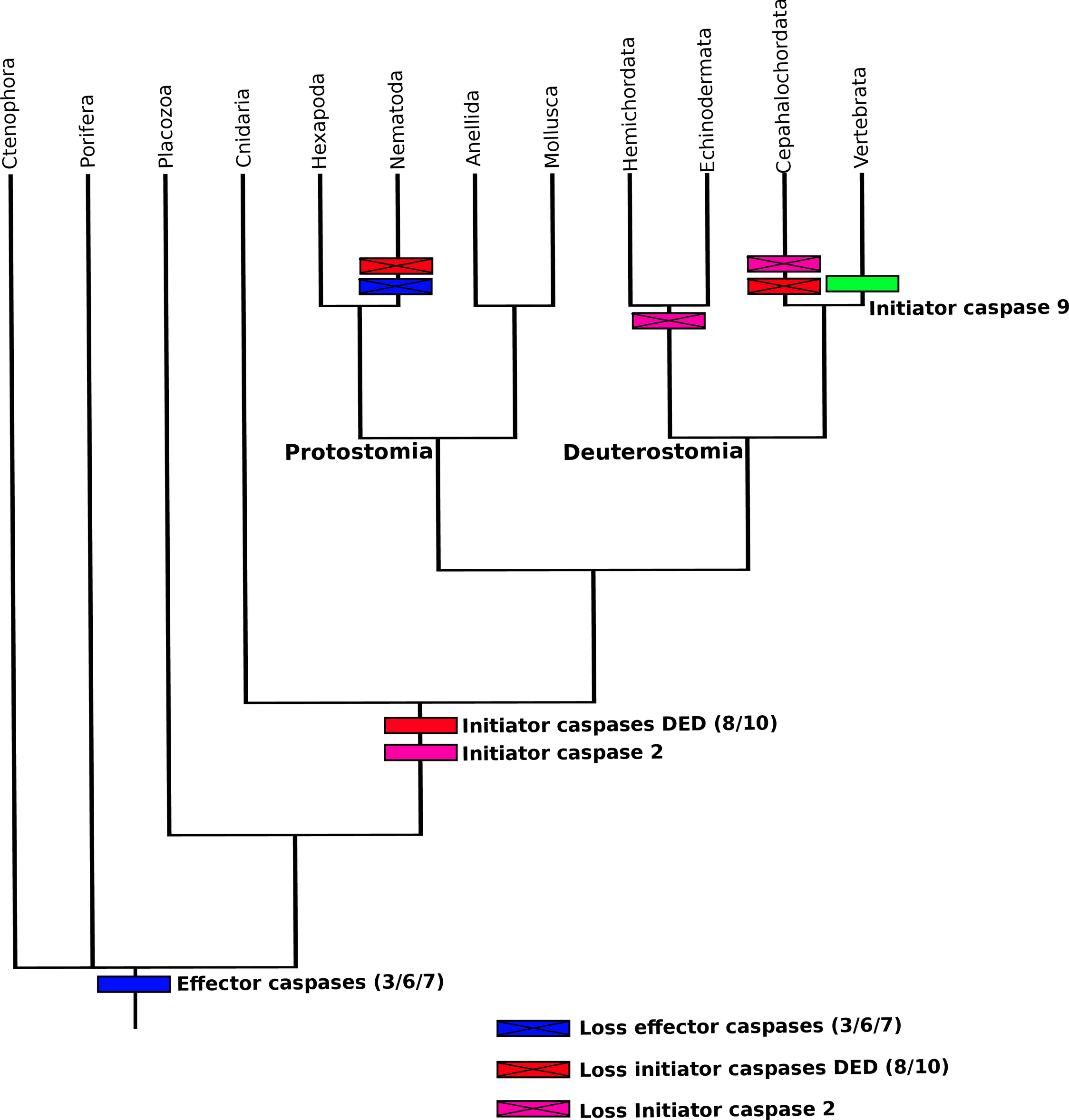

Supplement: S8 Fig — Initiator caspases appeared in the common ancestor of cnidarians and bilaterians but were subsequently lost independently in some groups (Nematoda, Hemichordata, Echinodermata and Cephalochordata). Caspase 9 is a specific acquisition of vertebrates. (TIF) [file ppat.1012320.s008.tif]

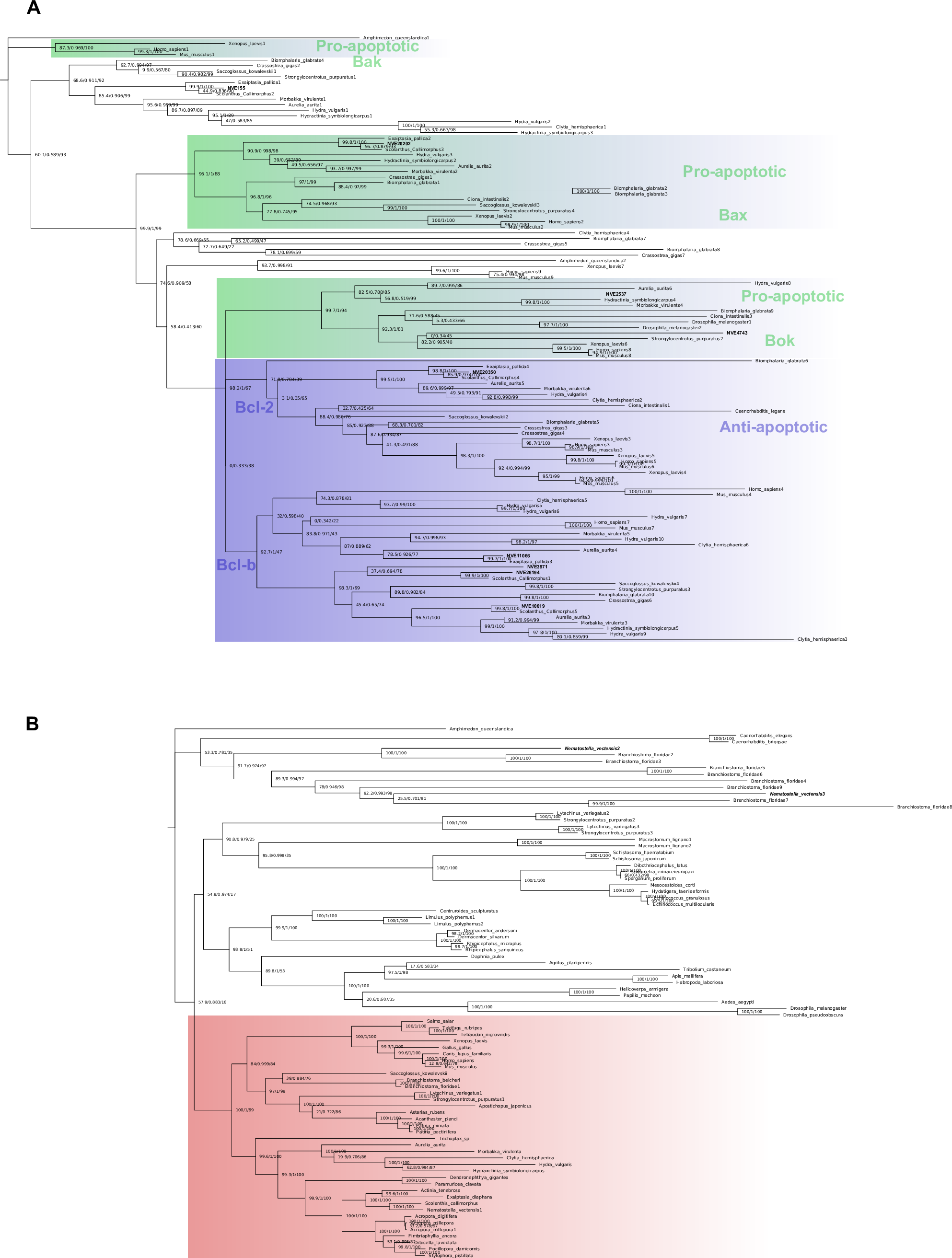

Supplement: S9 Fig — (A) Topology of the Bcl-2 family phylogeny determined by maximum likelihood at the metazoan scale. (B) Phylogeny of Apaf-1 across the metazoan scale, constructed using full-length sequence alignment. (TIF) [file ppat.1012320.s009.tif]
