# Supplementary material for: Induction of apoptosis by double-stranded RNA was present in the last common ancestor of cnidarian and bilaterian animals
Source: PLoS Pathog. 2024 Jul 16;20(7):e1012320. doi: 10.1371/journal.ppat.1012320 (PMC11251625; doi:10.1371/journal.ppat.1012320)
Supplement: S2 Table — (DOCX) [file ppat.1012320.s013.docx]

**Supplementary file 2:** Accession numbers for caspase, Apaf-1 and Bcl-2 protein sequences used in the BLAST search and phylogenetic analysis.

| **Name** | **Species** | **Genbank ID/Uniprot ID/Repository ID** |
| --- | --- | --- |
| Rfi_a | *Reticulomyxa filosa* | X6N4E9 |
| Rfi_b | *Reticulomyxa filosa* | X6NIS5 |
| Rfi_c | *Reticulomyxa filosa* | X6ME08 |
| Mle_casp3/6/7 | *Mnemiopsis leidyi* | <https://research.nhgri.nih.gov/mnemiopsis/download/proteome/ML2.2.aa.gz> (ML154125a) |
| Aqu_casp3a/6a/7a | *Amphimedon queenslandica* | A0A1X7VNG6 |
| Aqu_casp3b/6b/7b | *Amphimedon queenslandica* | A0A1X7UIG0 |
| Aqu_casp1-10/14 | *Amphimedon queenslandica* | <https://www.ncbi.nlm.nih.gov/datasets/genome/GCA_000090795.2> (XP_019854614.1) |
| Tsp_casp3/6/7 | *Trichoplax_sp* | A0A369SE24 |
| NVE23160_casp3/6/7 | *Nematostella vectensis* | A7S4V9 |
| NVE9681_casp8 | *Nematostella vectensis* | A7SPA7 |
| NVE5282_casp1-10/14 | *Nematostella vectensis* | GCF_932526225.1 |
| NVE20429_casp3/6/7 | *Nematostella vectensis* | A7S119 |
| NVE21851casp3/6/7 | *Nematostella vectensis* | A7S364 |
| NVE26090_casp1/2/4/5/14 | *Nematostella vectensis* | A7S9L1 |
| Sca_casp3a/6a/7a | *Scolanthus callimorphis* | <https://simrbase.stowers.org/files/pub/nematostella/Scal/genomes/Scal100/aligned/NY_Scal100_v1/NY_Scal100_v1.20200813.proteins.fasta> (NY_Scal100_v1.3230.1) |
| Sca_casp3c/6c/7c | *Scolanthus callimorphis* | <https://simrbase.stowers.org/files/pub/nematostella/Scal/genomes/Scal100/aligned/NY_Scal100_v1/NY_Scal100_v1.20200813.proteins.fasta> (NY_Scal100_v1.15401.1) |
| Sca_casp1-10/14 | *Scolanthus callimorphis* | <https://simrbase.stowers.org/files/pub/nematostella/Scal/genomes/Scal100/aligned/NY_Scal100_v1/NY_Scal100_v1.20200813.proteins.fasta> (NY_Scal100_v1.17616.1) |
| Sca_casp3b/6b/7b | *Scolanthus callimorphis* | <https://simrbase.stowers.org/files/pub/nematostella/Scal/genomes/Scal100/aligned/NY_Scal100_v1/NY_Scal100_v1.20200813.proteins.fasta> (NY_Scal100_v1.45819.1) |
| Sca_casp3d/6d/7d | *Scolanthus callimorphis* | <https://simrbase.stowers.org/files/pub/nematostella/Scal/genomes/Scal100/aligned/NY_Scal100_v1/NY_Scal100_v1.20200813.proteins.fasta> (NY_Scal100_v1.52845.1) |
| Sca_casp8 | *Scolanthus callimorphis* | <https://simrbase.stowers.org/files/pub/nematostella/Scal/genomes/Scal100/aligned/NY_Scal100_v1/NY_Scal100_v1.20200813.proteins.fasta> (NY_Scal100_v1.26481.1) |
| Sca_casp1/2/4/5/14 | *Scolanthus callimorphis* | <https://simrbase.stowers.org/files/pub/nematostella/Scal/genomes/Scal100/aligned/NY_Scal100_v1/NY_Scal100_v1.20200813.proteins.fasta> (NY_Scal100_v1.34496.1) |
| Ate_casp8 | *Actinia tenebrosa* | <https://www.ncbi.nlm.nih.gov/datasets/genome/GCF_009602425.1> (XP_031561863.1) |
| Ate_casp3b/6b/7b | *Actinia tenebrosa* | <https://www.ncbi.nlm.nih.gov/datasets/genome/GCF_009602425.1> (XP_031561639.1) |
| Ate_casp1-10/14 | *Actinia tenebrosa* | <https://www.ncbi.nlm.nih.gov/datasets/genome/GCF_009602425.1> (XP_031553730.1) |
| Ate_casp1/2/3/5/14 | *Actinia tenebrosa* | <https://www.ncbi.nlm.nih.gov/datasets/genome/GCF_009602425.1> (XP_031561638.1) |
| Ate_casp3c/6c/7c | *Actinia tenebrosa* | <https://www.ncbi.nlm.nih.gov/datasets/genome/GCF_009602425.1> (XP_031569424.1) |
| Ate_casp3a/6a/7a | *Actinia tenebrosa* | <https://www.ncbi.nlm.nih.gov/datasets/genome/GCF_009602425.1> (XP_031571668.1) |
| Spi_casp3d/6d/7d | *Stylophora pistillata* | A0A2B4RVA9 |
| Spi_casp3b/6b/7b | *Stylophora pistillata* | A0A2B4SJP8 |
| Spi_casp3a/6a/7a | *Stylophora pistillata* | A0A2B4RBN1 |
| Spi_casp8 | *Stylophora pistillata* | A0A2B4RVR5 |
| Spi_casp1/2/4/5/14 | *Stylophora pistillata* | A0A2B4SCW1 |
| Spi_casp3e/6e/7e | *Stylophora pistillata* | A0A2B4SWS7 |
| Spi_casp3c/6c/7c | *Stylophora pistillata* | A0A2B4SF88 |
| Adi_casp3d/6d/7d | *Acropora digitifera* | <https://www.ncbi.nlm.nih.gov/datasets/genome/GCF_000222465.1> (XP_015775441.1) |
| Adi_casp3c/6c/7c | *Acropora digitifera* | <https://www.ncbi.nlm.nih.gov/datasets/genome/GCF_000222465.1> (XP_015766400.1) |
| Adi_casp3a/6a/7a | *Acropora digitifera* | <https://www.ncbi.nlm.nih.gov/datasets/genome/GCF_000222465.1> (XP_015762375.1) |
| Adi_casp1-10/14 | *Acropora digitifera* | <https://www.ncbi.nlm.nih.gov/datasets/genome/GCF_000222465.1> (XP_015761120.1) |
| Adi_casp3b/6b/7b | *Acropora digitifera* | <https://www.ncbi.nlm.nih.gov/datasets/genome/GCF_000222465.1> (XP_015753767.1) |
| Adi_casp1/2/4/5/14 | *Acropora digitifera* | <https://www.ncbi.nlm.nih.gov/datasets/genome/GCF_000222465.1> (XP_015768208.1) |
| Edi_casp3b/6b/7b | *Exaiptasia diaphana* | <https://www.ncbi.nlm.nih.gov/datasets/genome/GCF_001417965.1> (XP_020903316.1) |
| Edi_casp1c/2c/4c/5c/14c | *Exaiptasia diaphana* | <https://www.ncbi.nlm.nih.gov/datasets/genome/GCF_001417965.1> (XP_020903315.1) |
| Edi_casp8 | *Exaiptasia diaphana* | <https://www.ncbi.nlm.nih.gov/datasets/genome/GCF_001417965.1> (XP_020892675.1) |
| Edi_casp3c/6c/7c | *Exaiptasia diaphana* | <https://www.ncbi.nlm.nih.gov/datasets/genome/GCF_001417965.1> (XP_020893866.1) |
| Edi_casp3a/6a/7a | *Exaiptasia diaphana* | <https://www.ncbi.nlm.nih.gov/datasets/genome/GCF_001417965.1> (XP_020905061.1) |
| Edi_casp1b/2b/4b/5b/14b | *Exaiptasia diaphana* | <https://www.ncbi.nlm.nih.gov/datasets/genome/GCF_001417965.1> (XP_028514208.1) |
| Edi_casp1a/2a/4a/5a/14a | *Exaiptasia diaphana* | <https://www.ncbi.nlm.nih.gov/datasets/genome/GCF_001417965.1> (XP_020897785.1) |
| Edi_casp1/4/5 | *Exaiptasia diaphana* | <https://www.ncbi.nlm.nih.gov/datasets/genome/GCF_001417965.1> (XP_028512718.1) |
| Che_casp3b/6b/7b | *Clytia hemisphaerica* | <https://research.nhgri.nih.gov/HydraAEP/download/sequences/c_hemi/clGen2.prot.fa.gz> (asmbl_46672) |
| Che_casp3a/6a/7a | *Clytia hemisphaerica* | <https://research.nhgri.nih.gov/HydraAEP/download/sequences/c_hemi/clGen2.prot.fa.gz> (asmbl_38047) |
| Che_casp8a | *Clytia hemisphaerica* | <https://research.nhgri.nih.gov/HydraAEP/download/sequences/c_hemi/clGen2.prot.fa.gz> (asmbl_10531) |
| Che_casp3c/6c/7c | *Clytia hemisphaerica* | <https://research.nhgri.nih.gov/HydraAEP/download/sequences/c_hemi/clGen2.prot.fa.gz> (asmbl_58488) |
| Che_casp8b | *Clytia hemisphaerica* | <https://research.nhgri.nih.gov/HydraAEP/download/sequences/c_hemi/clGen2.prot.fa.gz> (asmbl_33506) |
| Hvu_casp3c/6c/7c | *Hydra vulgaris* | D1MAR4 |
| Hvu_casp3b/6b/7b | *Hydra vulgaris* | Q9GV89 |
| Hvu_casp3e/6e/7e | *Hydra vulgaris* | A0A8B6XP53 |
| Hvu_casp3h/6h/7h | *Hydra vulgaris* | A0A8B6XQI4 |
| Hvu_casp3d/6d/7d | *Hydra vulgaris* | E2DGP9 |
| Hvu_casp3f/6f/7f | *Hydra vulgaris* | A0A8B6XIS7 |
| Hvu_casp8c | *Hydra vulgaris* | XP_047129731.1 |
| Hvu_casp8e | *Hydra vulgaris* | A0A8B7DUV5 |
| Hvu_casp8b | *Hydra vulgaris* | A0A8B7DEI2 |
| Hvu_casp8f | *Hydra vulgaris* | A0A8B7DAF8 |
| Hvu_casp8d | *Hydra vulgaris* | XP_047134542.1 |
| Hvu_casp3g/6g/7g | *Hydra vulgaris* | XP_047134596.1 |
| Hvu_casp3a/6a/7a | *Hydra vulgaris* | NP_001296709.1 |
| Hvu_casp8a | *Hydra vulgaris* | NP_001267753.1 |
| Hsy_casp3a/6a/7a | *Hydractinia symbiolongicarpus* | <https://research.nhgri.nih.gov/hydractinia/download/protein_models/symbio/Hsym_primary_v1.0.aa.gz> (HyS0023.328) |
| Hsy_casp3b/6b//7b | *Hydractinia symbiolongicarpus* | <https://research.nhgri.nih.gov/hydractinia/download/protein_models/symbio/Hsym_primary_v1.0.aa.gz> (HyS0012.28) |
| Hsy_casp3d/6d/7d | *Hydractinia symbiolongicarpus* | <https://research.nhgri.nih.gov/hydractinia/download/protein_models/symbio/Hsym_primary_v1.0.aa.gz> (HyS0031.82) |
| Hsy_casp3f/6f/7f | *Hydractinia symbiolongicarpus* | <https://research.nhgri.nih.gov/hydractinia/download/protein_models/symbio/Hsym_primary_v1.0.aa.gz> (HyS0074.45) |
| Hsy_casp3c/6c/7c | *Hydractinia symbiolongicarpus* | <https://research.nhgri.nih.gov/hydractinia/download/protein_models/symbio/Hsym_primary_v1.0.aa.gz> (HyS0003.486) |
| Hsy_casp3e/6e/7e | *Hydractinia symbiolongicarpus* | <https://research.nhgri.nih.gov/hydractinia/download/protein_models/symbio/Hsym_primary_v1.0.aa.gz> (HyS0034.180) |
| Mvi_casp3b/6b/7b | *Morbakka virulenta* | <https://marinegenomics.oist.jp/morbakka_virulenta/download/MOR05_r06_proteins.fa.gz> (scaffold222.g25.t1) |
| Mvi_casp8b | *Morbakka virulenta* | <https://marinegenomics.oist.jp/morbakka_virulenta/download/MOR05_r06_proteins.fa.gz> (scaffold618.g9.t1) |
| Mvi_casp3d/6d/7d | *Morbakka virulenta* | <https://marinegenomics.oist.jp/morbakka_virulenta/download/MOR05_r06_proteins.fa.gz> (scaffold244.g13.t1) |
| Mvi_casp3c/6c/7c | *Morbakka virulenta* | <https://marinegenomics.oist.jp/morbakka_virulenta/download/MOR05_r06_proteins.fa.gz> (scaffold222.g25.t2) |
| Mvi_casp8a | *Morbakka virulenta* | <https://marinegenomics.oist.jp/morbakka_virulenta/download/MOR05_r06_proteins.fa.gz> (scaffold618.g6.t1) |
| Mvi_casp3a/6a/7a | *Morbakka virulenta* | <https://marinegenomics.oist.jp/morbakka_virulenta/download/MOR05_r06_proteins.fa.gz> (scaffold213.g17.t1) |
| Aau_casp3b/6b/7b | *Aurelia aurita* | <https://marinegenomics.oist.jp/aurelia_aurita/download/AUR21_r04_proteins.fa.gz> (scaffold29.g89.t1) |
| Aau_casp8a | *Aurelia aurita* | <https://marinegenomics.oist.jp/aurelia_aurita/download/AUR21_r04_proteins.fa.gz> (scaffold67.g25.t1) |
| Aau_casp8b | *Aurelia aurita* | <https://marinegenomics.oist.jp/aurelia_aurita/download/AUR21_r04_proteins.fa.gz> (scaffold67.g16.t3) |
| Aau_casp3c/6c/7c | *Aurelia aurita* | <https://marinegenomics.oist.jp/aurelia_aurita/download/AUR21_r04_proteins.fa.gz> (scaffold29.g89.t2) |
| Aau_casp3d/6d/7d | *Aurelia aurita* | <https://marinegenomics.oist.jp/aurelia_aurita/download/AUR21_r04_proteins.fa.gz> (scaffold28.g80.t1) |
| Aau_casp3e/6e/7e | *Aurelia aurita* | <https://marinegenomics.oist.jp/aurelia_aurita/download/AUR21_r04_proteins.fa.gz> (scaffold20.g30.t1) |
| Aau_casp3a/6a/7a | *Aurelia aurita* | <https://marinegenomics.oist.jp/aurelia_aurita/download/AUR21_r04_proteins.fa.gz> (scaffold212.g26.t2) |
| Aau_casp8c | *Aurelia aurita* | <https://marinegenomics.oist.jp/aurelia_aurita/download/AUR21_r04_proteins.fa.gz> (scaffold37.g88.t1) |
| Cgi_casp2 | *Crassostrea gigas* | <https://www.ncbi.nlm.nih.gov/datasets/genome/GCA_902806645.1> (XP_011419292.2) |
| Cgi_casp1a/2a/4a/5a/14a | *Crassostrea gigas* | <https://www.ncbi.nlm.nih.gov/datasets/genome/GCA_902806645.1> (NP_001292299.1) |
| Cgi_casp10 | *Crassostrea gigas* | <https://www.ncbi.nlm.nih.gov/datasets/genome/GCA_902806645.1> (XP_034301971.1) |
| Cgi_casp1b/2b/4b/5b/14b | *Crassostrea gigas* | <https://www.ncbi.nlm.nih.gov/datasets/genome/GCA_902806645.1> (XP_011432762.2) |
| Cgi_casp3a/6a/7a | *Crassostrea gigas* | A0A8W8LCJ7 |
| Cgi_casp3b/6b/7b | *Crassostrea gigas* | <https://www.ncbi.nlm.nih.gov/datasets/genome/GCA_902806645.1> (XP_034310204.1) |
| Cgi_casp3/7 | *Crassostrea gigas* | <https://www.ncbi.nlm.nih.gov/datasets/genome/GCA_902806645.1> (XP_011445226.1) |
| Cte_casp1a/2a/4a/5a/14a | *Capitella teleta* | <https://www.ncbi.nlm.nih.gov/datasets/genome/GCA_000328365.1> (ELU15346.1) |
| Cte_casp3b/7b | *Capitella teleta* | <https://www.ncbi.nlm.nih.gov/datasets/genome/GCA_000328365.1> (ELU08285.1) |
| Cte_casp2 | *Capitella teleta* | <https://www.ncbi.nlm.nih.gov/datasets/genome/GCA_000328365.1> (ELT92962.1) |
| Cte_casp10 | *Capitella teleta* | <https://www.ncbi.nlm.nih.gov/datasets/genome/GCA_000328365.1> (ELU00616.1) |
| Cte_casp1b/2b/4b/5b/14b | *Capitella teleta* | <https://www.ncbi.nlm.nih.gov/datasets/genome/GCA_000328365.1> (ELT91797.1) |
| Cte_casp14 | *Capitella teleta* | <https://www.ncbi.nlm.nih.gov/datasets/genome/GCA_000328365.1> (ELT97848.1) |
| Cte_casp3a/7a | *Capitella teleta* | <https://www.ncbi.nlm.nih.gov/datasets/genome/GCA_000328365.1> (ELT90716.1) |
| Dme_casp1/2/4/5/14 | *Drosophila melanogaster* | Q9XYF4 |
| Dme_casp10 | *Drosophila melanogaster* | Q8IRY7 |
| Dme_casp3/6/7 | *Drosophila melanogaster* | Q7KHI6 |
| Dme_casp6b | *Drosophila melanogaster* | O02002 |
| Dme_casp6a | *Drosophila melanogaster* | Q9VET9 |
| Dme_casp6c | *Drosophila melanogaster* | O01382 |
| Cel_casp1a/4a/5a | *caenorhabditis elegans* | P42573 |
| Cel_casp1b/4b/5b | *caenorhabditis elegans* | Q9TZP5-3 |
| Cel_casp1c/4c/5c | *caenorhabditis elegans* | G5EBM1 |
| Dre_casp2 | *Danio rerio* | <https://www.ncbi.nlm.nih.gov/datasets/genome/GCA_000002035.4> (NP_001036160.1) |
| Dre_casp3/7 | *Danio rerio* | <https://www.ncbi.nlm.nih.gov/datasets/genome/GCA_000002035.4> (XP_001338890.2) |
| Dre_casp3 | *Danio rerio* | <https://www.ncbi.nlm.nih.gov/datasets/genome/GCA_000002035.4> (XP_005173132.1) |
| Dre_casp6b | *Danio rerio* | <https://www.ncbi.nlm.nih.gov/datasets/genome/GCA_000002035.4> (XP_005164109.1) |
| Dre_casp6c | *Danio rerio* | <https://www.ncbi.nlm.nih.gov/datasets/genome/GCA_000002035.4> (XP_017210076.1) |
| Dre_casp6a | *Danio rerio* | <https://www.ncbi.nlm.nih.gov/datasets/genome/GCA_000002035.4> NP_001018333.1) |
| Dre_casp7 | *Danio rerio* | <https://www.ncbi.nlm.nih.gov/datasets/genome/GCA_000002035.4> (XP_005156389.1) |
| Dre_casp10a | *Danio rerio* | <https://www.ncbi.nlm.nih.gov/datasets/genome/GCA_000002035.4> (XP_005165894.1) |
| Dre_casp10b | *Danio rerio* | <https://www.ncbi.nlm.nih.gov/datasets/genome/GCA_000002035.4> (NP_001092089.1) |
| Dre_casp9 | *Danio rerio* | <https://www.ncbi.nlm.nih.gov/datasets/genome/GCA_000002035.4> (NP_001007405.2) |
| Hsa_casp1 | *Homo sapiens* | P29466 |
| Hsa_casp2 | *Homo sapiens* | P42575 |
| Hsa_casp3 | *Homo sapiens* | P42574 |
| Hsa_casp4 | *Homo sapiens* | P49662 |
| Hsa_casp5 | *Homo sapiens* | P51878 |
| Hsa_casp8 | *Homo sapiens* | O15519 |
| Hsa_casp14 | *Homo sapiens* | P31944 |
| Hsa_casp7 | *Homo sapiens* | P55210-3 |
| Hsa_casp9 | *Homo sapiens* | P55211 |
| Hsa_casp6 | *Homo sapiens* | P55212 |
| Hsa_casp10 | *Homo sapiens* | Q92851 |
| Mmu_casp1 | *Mus musculus* | P29452 |
| Mmu_casp2 | *Mus musculus* | P29594 |
| Mmu_casp3 | *Mus musculus* | P70677 |
| Mmu_casp6 | *Mus musculus* | O08738 |
| Mmu_casp7 | *Mus musculus* | P97864 |
| Mmu_casp10 | *Mus musculus* | O89110 |
| Mmu_casp9 | *Mus musculus* | Q8C3Q9 |
| Mmu_casp4b/5b | *Mus musculus* | P70343 |
| Mmu_casp4a/5a | *Mus musculus* | O08736 |
| Mmu_casp14 | *Mus musculus* | O89094 |
| Spu_casp3d/6d/7d | *Strongylocentrotus purpuratus* | <https://www.ncbi.nlm.nih.gov/datasets/genome/GCA_000002235.4> (XP_030842855.1) |
| Spu_casp3e/6e/7e | *Strongylocentrotus purpuratus* | <https://www.ncbi.nlm.nih.gov/datasets/genome/GCA_000002235.4> (XP_030843307.1) |
| Spu_casp1b/4b/5b | *Strongylocentrotus purpuratus* | <https://www.ncbi.nlm.nih.gov/datasets/genome/GCA_000002235.4> (XP_030836939.1) |
| Spu_casp1c/4c/5c | *Strongylocentrotus purpuratus* | <https://www.ncbi.nlm.nih.gov/datasets/genome/GCA_000002235.4> (XP_788060.1) |
| Spu_casp3a/6a/7a | *Strongylocentrotus purpuratus* | <https://www.ncbi.nlm.nih.gov/datasets/genome/GCA_000002235.4> (XP_030834949.1) |
| Spu_casp3b/6b/7b | *Strongylocentrotus purpuratus* | <https://www.ncbi.nlm.nih.gov/datasets/genome/GCA_000002235.4> (XP_030842852.1) |
| Spu_casp6 | *Strongylocentrotus purpuratus* | <https://www.ncbi.nlm.nih.gov/datasets/genome/GCA_000002235.4> (XP_030843308.1) |
| Spu_casp3/7 | *Strongylocentrotus purpuratus* | <https://www.ncbi.nlm.nih.gov/datasets/genome/GCA_000002235.4> (XP_030851381.1) |
| Spu_casp1a/4a/5a | *Strongylocentrotus purpuratus* | <https://www.ncbi.nlm.nih.gov/datasets/genome/GCA_000002235.4> (XP_030843171.1) |
| Spu_casp3c/6c/7c | *Strongylocentrotus purpuratus* | <https://www.ncbi.nlm.nih.gov/datasets/genome/GCA_000002235.4> (XP_030843308.1) |
| Spu_casp10 | *Strongylocentrotus purpuratus* | <https://www.ncbi.nlm.nih.gov/datasets/genome/GCA_000002235.4> (XP_030830139.1) |
| Sko_casp3a/7a | *Saccoglossus kowalevskii* | <https://www.ncbi.nlm.nih.gov/datasets/genome/GCA_000003605.1> (XP_002730721.1) |
| Sko_casp3c/7c | *Saccoglossus kowalevskii* | <https://www.ncbi.nlm.nih.gov/datasets/genome/GCA_000003605.1> (XP_002738921.1) |
| Sko_casp3d/7d | *Saccoglossus kowalevskii* | <https://www.ncbi.nlm.nih.gov/datasets/genome/GCA_000003605.1> (XP_006821694.1) |
| Sko_casp3/6/7 | *Saccoglossus kowalevskii* | <https://www.ncbi.nlm.nih.gov/datasets/genome/GCA_000003605.1> (XP_006811879.1) |
| Sko_casp10b | *Saccoglossus kowalevskii* | <https://www.ncbi.nlm.nih.gov/datasets/genome/GCA_000003605.1> (XP_006816014.1) |
| Sko_casp6 | *Saccoglossus kowalevskii* | <https://www.ncbi.nlm.nih.gov/datasets/genome/GCA_000003605.1> (XP_002737264.1) |
| Sko_casp3b/7b | *Saccoglossus kowalevskii* | <https://www.ncbi.nlm.nih.gov/datasets/genome/GCA_000003605.1> (XP_002734878.2) |
| Sko_casp10a | *Saccoglossus kowalevskii* | <https://www.ncbi.nlm.nih.gov/datasets/genome/GCA_000003605.1> (XP_002739483.1) |
| Sko_casp10c | *Saccoglossus kowalevskii* | <https://www.ncbi.nlm.nih.gov/datasets/genome/GCA_000003605.1> (XP_002732925.1) |
| Bbe_casp3/6/7 | *Branchiostoma belcheri* | A0A6P5AKF3 |
| Bbe_casp3/7 | *Branchiostoma belcheri* | A0A6P4ZZL3 |

| **Name** | **Species** | **Genbank ID/Uniprot ID** |
| --- | --- | --- |
| Amphimedon_queenslandica | *Amphimedon queenslandica* | XP_019855714.1 |
| Trichoplax_sp | *Trichoplax_sp* | RDD40813.1 |
| Acropora digitifera | *Acropora digitifera* | XP_015776356.1 |
| Acropora_millepora | *Acropora millepora* | AJG37574.1 |
| Acropora_millepora1 | *Acropora millepora* | XP_029185121.1 |
| Actinia_tenebrosa | *Actinia tenebrosa* | XP_031572908.1 |
| Dendronephthya_gigantea | *Dendronephthya gigantea* | XP_028393942.1 |
| Exaiptasia_diaphana | *Exaiptasia diaphana* | KXJ17575.1 |
| Fimbriaphyllia_ancora | *Fimbriaphyllia ancora* | QMS47765.1 |
| Hydra_vulgaris | *Hydra_vulgaris* | CDG72123.1 |
| Clytia_hemisphaerica | *Clytia hemisphaerica* | <https://research.nhgri.nih.gov/HydraAEP/download/sequences/c_hemi/clGen2.prot.fa.gz> (asmbl_48366) |
| Hydractinia_symbiolongicarpus | *Hydractinia symbiolongicarpus* | <https://research.nhgri.nih.gov/hydractinia/download/protein_models/symbio/Hsym_primary_v1.0.aa.gz> (HyS0046.129) |
| Morbakka_virulenta | *Morbakka virulenta* | <https://marinegenomics.oist.jp/morbakka_virulenta/download/MOR05_r06_proteins.fa.gz> (scaffold323.g44.t2) |
| Scolanthus_callimorphus | *Scolanthus callimorphus* | <https://simrbase.stowers.org/files/pub/nematostella/Scal/genomes/Scal100/aligned/NY_Scal100_v1/NY_Scal100_v1.20200813.proteins.fasta> (NY_Scal100_v1.6927.1) |
| Nematostella_vectensis1 | *Nematostella vectensis* | XP_032220711.2 |
| Nematostella_vectensis2 | *Nematostella vectensis* | XP_032223290.2 |
| Nematostella_vectensis3 | *Nematostella vectensis* | XP_032225984.2 |
| Orbicella_faveolata | *Orbicella faveolata* | XP_020620792.1 |
| Paramuricea_clavata | *Paramuricea clavata* | CAB4025087.1 |
| Pocillopora_damicornis | *Pocillopora_damicornis* | XP_027042245.1 |
| Stylophora_pistillata | *Stylophora_pistillata* | XP_022790799.1 |
| Aurelia_aurita | *Aurelia aurita* | <https://marinegenomics.oist.jp/aurelia_aurita/download/AUR21_r04_proteins.fa.gz> (scaffold58.g1.t1) |
| Caenorhabditis_elegans | *Caenorhabditis elegans* | CAA48781.1 |
| Caenorhabditis_briggsae | *Caenorhabditis briggsae* | XP_002642027.2 |
| Aedes_aegypti | *Aedes aegypti* | EAT48066.2 |
| Agrilus_planipennis | *Agrilus planipennis* | XP_025834330.1 |
| Apis_mellifera | *Apis mellifera* | XP_006565907.1 |
| Drosophila_melanogaster | *Drosophila melanogaster* | NP_725637.1 |
| Drosophila_pseudoobscura | *Drosophila pseudoobscura* | XP_001360832.3 |
| Habropoda_laboriosa | *Habropoda laboriosa* | XP_017794472.1 |
| Helicoverpa_armigera | *Helicoverpa armigera* | XP_021181657.1 |
| Papilio_machaon | *Papilio machaon* | XP_014359375.1 |
| Tribolium_castaneum | *Tribolium castaneum* | XP_015840766.1 |
| Daphnia_pulex | *Daphnia pulex* | XP_046440096.1 |
| Centruroides_sculpturatus | *Centruroides sculpturatus* | XP_023242710.1 |
| Dermacentor_andersoni | *Dermacentor andersoni* | XP_050040075.1 |
| Dermacentor_silvarum | *Dermacentor silvarum* | XP_037568890.1 |
| Limulus_polyphemus1 | *Limulus polyphemus* | XP_022249946.1 |
| Limulus_polyphemus2 | *Limulus polyphemus* | XP_022244405.1 |
| Rhipicephalus_microplus | *Rhipicephalus microplus* | XP_037276898.1 |
| Rhipicephalus_sanguineus | *Rhipicephalus sanguineus* | XP_037506725.1 |
| Dibothriocephalus_latus | *Dibothriocephalus latus* | VDN16554.1 |
| Echinococcus_granulosus | *Echinococcus granulosus* | XP_024354545.1 |
| Echinococcus_multilocularis | *Echinococcus multilocularis* | CDI98465.1 |
| Hydatigera_taeniaeformis | *Hydatigera taeniaeformis* | VDM18691.1 |
| Macrostomum_lignano1 | *Macrostomum lignano* | PAA53960.1 |
| Macrostomum_lignano2 | *Macrostomum lignano* | PAA76335.1 |
| Mesocestoides_corti | *Mesocestoides corti* | VDD80977.1 |
| Schistosoma_haematobium | *Schistosoma haematobium* | XP_035588882.1 |
| Schistosoma_japonicum | *Schistosoma japonicum* | KAH8853391.1 |
| Spirometra_erinaceieuropaei | *Spirometra erinaceieuropaei* | VZI41625.1 |
| Sparganum_proliferum | *Sparganum proliferum* | VZI22297.1 |
| Homo_sapiens | *Homo sapiens* | XP_047284715.1 |
| Mus_musculus | *Mus musculus* | NP_033814.2 |
| Canis_lupus_familiaris | *Canis lupus familiaris* | XP_038544278.1 |
| Salmo_salar | *Salmo salar* | XP_014063984.1 |
| Gallus_gallus | *Gallus gallus* | XP_040518239.1 |
| Xenopus_laevis | *Xenopus laevis* | NP_001085834.1 |
| Takifugu_rubripes | *Takifugu rubripes* | XP_011611631.2 |
| Tetraodon_nigroviridis | *Tetraodon nigroviridis* | CAG01878.1 |
| Acanthaster_planci | *Acanthaster planci* | XP_022083681.1 |
| Apostichopus_japonicus | *Apostichopus japonicus* | PIK57251.1 |
| Asterias_rubens | *Asterias rubens* | XP_033645922.1 |
| Lytechinus_variegatus1 | *Lytechinus variegatus* | XP_041472197.1 |
| Lytechinus_variegatus2 | *Lytechinus variegatus* | XP_041469796.1 |
| Lytechinus_variegatus3 | *Lytechinus variegatus* | XP_041459069.1 |
| Patiria_miniata | *Patiria miniata* | XP_038054179.1 |
| Patiria_pectinifera | *Patiria pectinifera* | AUQ44343.1 |
| Strongylocentrotus_purpuratus1 | *Strongylocentrotus purpuratus* | XP_030844937.1 |
| Strongylocentrotus_purpuratus2 | *Strongylocentrotus purpuratus* | XP_030854919.1 |
| Strongylocentrotus_purpuratus3 | *Strongylocentrotus purpuratus* | XP_030828596.1 |
| Saccoglossus_kowalevskii | *Saccoglossus kowalevskii* | XP_006818297.1 |
| Branchiostoma_belcheri | *Branchiostoma belcheri* | XP_019621685.1 |
| Branchiostoma_floridae1 | *Branchiostoma belcheri* | XP_035681983.1 |
| Branchiostoma_floridae2 | *Branchiostoma belcheri* | XP_035678916.1 |
| Branchiostoma_floridae3 | *Branchiostoma belcheri* | XP_035672288.1 |
| Branchiostoma_floridae4 | *Branchiostoma belcheri* | XP_035690210.1 |
| Branchiostoma_floridae5 | *Branchiostoma belcheri* | XP_035667061.1 |
| Branchiostoma_floridae6 | *Branchiostoma belcheri* | XP_035696241.1 |
| Branchiostoma_floridae7 | *Branchiostoma belcheri* | XP_035693308.1 |
| Branchiostoma_floridae8 | *Branchiostoma belcheri* | XP_035673101.1 |
| Branchiostoma_floridae9 | *Branchiostoma belcheri* | XP_035692718.1 |

| **Name** | **Species** | **Genbank ID/Uniprot ID** |
| --- | --- | --- |
| Amphimedon_queenslandica1 | *Amphimedon queenslandica* | XP_003383425.1 |
| Amphimedon_queenslandica2 | *Amphimedon queenslandica* | XP_003387574.1 |
| Hydra_vulgaris1 | *Hydra vulgaris* | ABL01492.1 |
| Hydra_vulgaris2 | *Hydra vulgaris* | ABS84169.1 |
| Hydra_vulgaris3 | *Hydra vulgaris* | XP_012562061.1 |
| Hydra_vulgaris4 | *Hydra vulgaris* | ABS84173.1 |
| Hydra_vulgaris5 | *Hydra vulgaris* | ABL01493.1 |
| Hydra_vulgaris6 | *Hydra vulgaris* | ABS84174.1 |
| Hydra_vulgaris7 | *Hydra vulgaris* | ABS84172.1 |
| Hydra_vulgaris8 | *Hydra vulgaris* | ABS84170.1 |
| Hydra_vulgaris9 | *Hydra vulgaris* | ABL01494.1 |
| Hydra_vulgaris10 | *Hydra vulgaris* | ABS84171.1 |
| NVE155 | *Nematostella vectensis* | GCF_932526225.1 |
| NVE26194 | *Nematostella vectensis* | GCF_932526225.1 |
| NVE10019 | *Nematostella vectensis* | A7SPR2 |
| NVE20350 | *Nematostella vectensis* | A7S109 |
| NVE11066 | *Nematostella vectensis* | A7SRF5 |
| NVE20202 | *Nematostella vectensis* | A7S0T4 |
| NVE3971 | *Nematostella vectensis* | GCF_932526225.1 |
| NVE2537 | *Nematostella vectensis* | A7S839 |
| NVE4743 | *Nematostella vectensis* | A7RN20 |
| Aurelia_aurita1 | *Aurelia aurita* | <https://marinegenomics.oist.jp/aurelia_aurita/download/AUR21_r04_proteins.fa.gz> (scaffold29.g36.t1) |
| Aurelia_aurita2 | *Aurelia aurita* | <https://marinegenomics.oist.jp/aurelia_aurita/download/AUR21_r04_proteins.fa.gz> (scaffold153.g43.t1) |
| Aurelia_aurita3 | *Aurelia aurita* | <https://marinegenomics.oist.jp/aurelia_aurita/download/AUR21_r04_proteins.fa.gz> (scaffold12.g95.t1) |
| Aurelia_aurita4 | *Aurelia aurita* | <https://marinegenomics.oist.jp/aurelia_aurita/download/AUR21_r04_proteins.fa.gz> (scaffold274.g16.t2) |
| Aurelia_aurita5 | *Aurelia aurita* | <https://marinegenomics.oist.jp/aurelia_aurita/download/AUR21_r04_proteins.fa.gz> (scaffold29.g40.t1) |
| Aurelia_aurita6 | *Aurelia aurita* | <https://marinegenomics.oist.jp/aurelia_aurita/download/AUR21_r04_proteins.fa.gz> (scaffold109.g8.t1) |
| Clytia_hemisphaerica1 | *Clytia hemisphaerica* | GCA_902728285.1 |
| Clytia_hemisphaerica2 | *Clytia hemisphaerica* | <https://research.nhgri.nih.gov/HydraAEP/download/sequences/c_hemi/clGen2.prot.fa.gz> (asmbl_36347) |
| Clytia_hemisphaerica3 | *Clytia hemisphaerica* | <https://research.nhgri.nih.gov/HydraAEP/download/sequences/c_hemi/clGen2.prot.fa.gz> (asmbl_5744) |
| Clytia_hemisphaerica4 | *Clytia hemisphaerica* | GCA_902728285.1 |
| Clytia_hemisphaerica5 | *Clytia hemisphaerica* | <https://research.nhgri.nih.gov/HydraAEP/download/sequences/c_hemi/clGen2.prot.fa.gz> (asmbl_51873) |
| Clytia_hemisphaerica6 | *Clytia hemisphaerica* | <https://research.nhgri.nih.gov/HydraAEP/download/sequences/c_hemi/clGen2.prot.fa.gz> (asmbl_1721) |
| Hydractinia_symbiolongicarpus1 | *Hydractinia symbiolongicarpus* | <https://research.nhgri.nih.gov/hydractinia/download/protein_models/symbio/Hsym_primary_v1.0.aa.gz> (HyS0005.96) |
| Hydractinia_symbiolongicarpus2 | *Hydractinia symbiolongicarpus* | <https://research.nhgri.nih.gov/hydractinia/download/protein_models/symbio/Hsym_primary_v1.0.aa.gz> (HyS0052.122) |
| Hydractinia_symbiolongicarpus3 | *Hydractinia symbiolongicarpus* | <https://research.nhgri.nih.gov/hydractinia/download/protein_models/symbio/Hsym_primary_v1.0.aa.gz> (HyS0005.94) |
| Hydractinia_symbiolongicarpus4 | *Hydractinia symbiolongicarpus* | <https://research.nhgri.nih.gov/hydractinia/download/protein_models/symbio/Hsym_primary_v1.0.aa.gz> (HyS0001.229) |
| Hydractinia_symbiolongicarpus5 | *Hydractinia symbiolongicarpus* | <https://research.nhgri.nih.gov/hydractinia/download/protein_models/symbio/Hsym_primary_v1.0.aa.gz> (HyS0007.62) |
| Morbakka_virulenta1 | *Morbakka virulenta* | <https://marinegenomics.oist.jp/morbakka_virulenta/download/MOR05_r06_proteins.fa.gz> (scaffold128.g2.t1) |
| Morbakka_virulenta2 | *Morbakka virulenta* | <https://marinegenomics.oist.jp/morbakka_virulenta/download/MOR05_r06_proteins.fa.gz> (scaffold151.g39.t1) |
| Morbakka_virulenta3 | *Morbakka virulenta* | <https://marinegenomics.oist.jp/morbakka_virulenta/download/MOR05_r06_proteins.fa.gz> (scaffold143.g21.t1) |
| Morbakka_virulenta4 | *Morbakka virulenta* | <https://marinegenomics.oist.jp/morbakka_virulenta/download/MOR05_r06_proteins.fa.gz> (scaffold345.g18.t1) |
| Morbakka_virulenta5 | *Morbakka virulenta* | <https://marinegenomics.oist.jp/morbakka_virulenta/download/MOR05_r06_proteins.fa.gz> (scaffold19.g11.t1) |
| Morbakka_virulenta6 | *Morbakka virulenta* | <https://marinegenomics.oist.jp/morbakka_virulenta/download/MOR05_r06_proteins.fa.gz> (scaffold462.g9.t1) |
| Saccoglossus_kowalevskii1 | *Saccoglossus kowalevskii* | <https://www.ncbi.nlm.nih.gov/datasets/genome/GCA_000003605.1> (XP_006812549.1) |
| Saccoglossus_kowalevskii2 | *Saccoglossus kowalevskii* | <https://www.ncbi.nlm.nih.gov/datasets/genome/GCA_000003605.1> (XP_002740789.2) |
| Saccoglossus_kowalevskii3 | *Saccoglossus kowalevskii* | <https://www.ncbi.nlm.nih.gov/datasets/genome/GCA_000003605.1> (XP_006822173.1) |
| Saccoglossus_kowalevskii4 | *Saccoglossus kowalevskii* | <https://www.ncbi.nlm.nih.gov/datasets/genome/GCA_000003605.1> (XP_002730739.1) |
| Exaiptasia_pallida1 | *Exaiptasia diaphana* | <https://www.ncbi.nlm.nih.gov/datasets/genome/GCF_001417965.1> (XP_020902779.1) |
| Exaiptasia_pallida2 | *Exaiptasia diaphana* | <https://www.ncbi.nlm.nih.gov/datasets/genome/GCF_001417965.1> (XP_020906977.1) |
| Exaiptasia_pallida3 | *Exaiptasia diaphana* | <https://www.ncbi.nlm.nih.gov/datasets/genome/GCF_001417965.1> (XP_028512709.1) |
| Exaiptasia_pallida4 | *Exaiptasia diaphana* | <https://www.ncbi.nlm.nih.gov/datasets/genome/GCF_001417965.1> (XP_020912229.1) |
| Scolanthus_Callimorphus1 | *Scolanthus callimorphus* | <https://simrbase.stowers.org/files/pub/nematostella/Scal/genomes/Scal100/aligned/NY_Scal100_v1/NY_Scal100_v1.20200813.proteins.fasta> (NY_Scal100_v1.21614.1) |
| Scolanthus_Callimorphus2 | *Scolanthus callimorphus* | <https://simrbase.stowers.org/files/pub/nematostella/Scal/genomes/Scal100/aligned/NY_Scal100_v1/NY_Scal100_v1.20200813.proteins.fasta> (NY_Scal100_v1.781.1) |
| Scolanthus_Callimorphus3 | *Scolanthus callimorphus* | <https://simrbase.stowers.org/files/pub/nematostella/Scal/genomes/Scal100/aligned/NY_Scal100_v1/NY_Scal100_v1.20200813.proteins.fasta> (NY_Scal100_v1.16199.1) |
| Scolanthus_Callimorphus4 | *Scolanthus callimorphus* | <https://simrbase.stowers.org/files/pub/nematostella/Scal/genomes/Scal100/aligned/NY_Scal100_v1/NY_Scal100_v1.20200813.proteins.fasta> (NY_Scal100_v1.28731.1) |
| Scolanthus_Callimorphus5 | *Scolanthus callimorphus* | <https://simrbase.stowers.org/files/pub/nematostella/Scal/genomes/Scal100/aligned/NY_Scal100_v1/NY_Scal100_v1.20200813.proteins.fasta> (NY_Scal100_v1.6145.1) |
| Ciona_intestinalis1 | *Ciona intestinalis* | <https://www.ncbi.nlm.nih.gov/datasets/genome/GCA_000224145.2> (XP_002127107.1) |
| Ciona_intestinalis2 | *Ciona intestinalis* | <https://www.ncbi.nlm.nih.gov/datasets/genome/GCA_000224145.2> (XP_002123003.1) |
| Ciona_intestinalis3 | *Ciona intestinalis* | <https://www.ncbi.nlm.nih.gov/datasets/genome/GCA_000224145.2> (XP_026695029.1) |
| Strongylocentrotus_purpuratus1 | *Strongylocentrotus purpuratus* | <https://www.ncbi.nlm.nih.gov/datasets/genome/GCA_000002235.4> (XP_030843836.1) |
| Strongylocentrotus_purpuratus2 | *Strongylocentrotus purpuratus* | <https://www.ncbi.nlm.nih.gov/datasets/genome/GCA_000002235.4> (XP_030837754.1) |
| Strongylocentrotus_purpuratus3 | *Strongylocentrotus purpuratus* | <https://www.ncbi.nlm.nih.gov/datasets/genome/GCA_000002235.4> (XP_787479.3) |
| Strongylocentrotus_purpuratus4 | *Strongylocentrotus purpuratus* | <https://www.ncbi.nlm.nih.gov/datasets/genome/GCA_000002235.4> (XP_030844983.1) |
| Caenorhabditis_legans | *Caenorhabditis elegans* | AAA20080.1 |
| Drosophila_melanogaster1 | *Drosophila melanogaster* | AAF44120.1 |
| Drosophila_melanogaster2 | *Drosophila melanogaster* | AAF26289.1 |
| Biomphalaria_glabrata1 | *Biomphalaria glabrata* | XP_013085524.1 |
| Biomphalaria_glabrata2 | *Biomphalaria glabrata* | XP_013096338.2 |
| Biomphalaria_glabrata3 | *Biomphalaria glabrata* | XP_013086802.1 |
| Biomphalaria_glabrata4 | *Biomphalaria glabrata* | XP_013070177.1 |
| Biomphalaria_glabrata5 | *Biomphalaria glabrata* | XP_013081872.1 |
| Biomphalaria_glabrata6 | *Biomphalaria glabrata* | XP_013093137.1 |
| Biomphalaria_glabrata7 | *Biomphalaria glabrata* | XP_013068612.1 |
| Biomphalaria_glabrata8 | *Biomphalaria glabrata* | XP_013083436.1 |
| Biomphalaria_glabrata9 | *Biomphalaria glabrata* | XP_013069706.1 |
| Biomphalaria_glabrata10 | *Biomphalaria glabrata* | XP_013065969.1 |
| Crassostrea_gigas1 | *Crassostrea gigas* | XP_011424481.1 |
| Crassostrea_gigas2 | *Crassostrea gigas* | XP_011439700.1 |
| Crassostrea_gigas3 | *Crassostrea gigas* | XP_011449013.1 |
| Crassostrea_gigas4 | *Crassostrea gigas* | ACH42081.1 |
| Crassostrea_gigas5 | *Crassostrea gigas* | EKC18663.1 |
| Crassostrea_gigas6 | *Crassostrea gigas* | XP_011436990.1 |
| Crassostrea_gigas7 | *Crassostrea gigas* | EKC40007.1 |
| Homo_sapiens1 | *Homo sapiens* | AAA74466.1 |
| Homo_sapiens2 | *Homo sapiens* | NP_001278357.1 |
| Homo_sapiens3 | *Homo sapiens* | API71171.1 |
| Homo_sapiens4 | *Homo sapiens* | AAK48715.1 |
| Homo_sapiens5 | *Homo sapiens* | AAB09055.1 |
| Homo_sapiens6 | *Homo sapiens* | CAA80661.1 |
| Homo_sapiens7 | *Homo sapiens* | NP_004040.1 |
| Homo_sapiens8 | *Homo sapiens* | NP_115904.1 |
| Homo_sapiens9 | *Homo sapiens* | AAF64255.1 |
| Mus_musculus1 | *Mus musculus* | NP_031549.2 |
| Mus_musculus2 | *Mus musculus* | NP_031553.1 |
| Mus_musculus3 | *Mus musculus* | AAH95964.1 |
| Mus_musculus4 | *Mus musculus* | Q9Z0F3.1 |
| Mus_musculus5 | *Mus musculus* | AAA51039.1 |
| Mus_musculus6 | *Mus musculus* | AAB09056.1 |
| Mus_musculus7 | *Mus musculus* | Q07440.1 |
| Mus_musculus8 | *Mus musculus* | NP_058058.1 |
| Mus_musculus9 | *Mus musculus* | NP_032588.1 |
| Xenopus_laevis1 | *Xenopus laevis* | NP_001089587.1 |
| Xenopus_laevis2 | *Xenopus laevis* | AAR84081.1 |
| Xenopus_laevis3 | *Xenopus laevis* | BAH28834.1 |
| Xenopus_laevis4 | *Xenopus laevis* | AAI10791.1 |
| Xenopus_laevis5 | *Xenopus laevis* | XP_018089640.1 |
| Xenopus_laevis6 | *Xenopus laevis* | NP_001139563.1 |
| Xenopus_laevis7 | *Xenopus laevis* | ACI47310.1 |
