# Supplementary material for: Induction of apoptosis by double-stranded RNA was present in the last common ancestor of cnidarian and bilaterian animals
Source: PLoS Pathog. 2024 Jul 16;20(7):e1012320. doi: 10.1371/journal.ppat.1012320 (PMC11251625; doi:10.1371/journal.ppat.1012320)
Supplement: Attachment — Submitted filename: Response letter.pdf [file ppat.1012320.s019.pdf]

Jerusalem, May 9, 2024

Dear Editors,

We are grateful for the time and effort you as well as the three reviewers spent on handling and reviewing our manuscript. We felt that all the comments were thoughtful and very helpful in improving our paper. In addition to a copy of our revised manuscript where we clearly marked all changes we also provide below a point-by-point response to the specific comments made by the three reviewers. We hope that you will find our revision satisfactory and suitable for acceptance to *PLOS Pathogens*.

Many thanks and kind regards,
Yehu Moran and Itamar Kozlovski on behalf of all authors

Part II – Major Issues: Key Experiments Required for Acceptance
Please use this section to detail the key new experiments or modifications of existing experiments that should be absolutely required to validate study conclusions.

Generally, there should be no more than 3 such required experiments or major modifications for a "Major Revision" recommendation. If more than 3 experiments are necessary to validate the study conclusions, then you are encouraged to recommend "Reject".

Reviewer 1

1. Reviewer comment: The experimental design for some of the experiments is confusing (Figure 1,2). Because of potential variance between experiments, I would expect a consistent positive control with apoptosis inducers across the experiments to account for the wide variance in the arbitrary units of fluorescence. Can it be that the Mock control already induces apoptosis? Please explain the design. A positive control is also particularly relevant for the detection in the cas-3 assay which in some time points exhibit a high variance compounded with only 3 replicates.

Response to reviewer:

Thank you for this constructive comment. Regarding the experimental design, by mock transfection we mean the same formulation of lipofectamine as the one used in poly(I:C) transfection, without the poly(I:C). This is now mentioned clearly in methods section. Indeed, it is possible that lipofectamine alone exerted some toxicity. Such toxicity was previously described in mammalian cell lines (https://www.ncbi.nlm.nih.gov/pmc/articles/PMC6222501/) and it is therefore essential to compare the effect of poly(I:C) to the lipofectamine (mock) control. Similarly, in the *in vivo* experiments we used the same solution of NaCl as the one used for microinjection of poly(I:C). Regarding a positive control, we now included mitomycin-c in both caspase-3 activity (**S6 Fig.**) assays and in Apotracker Green assays (**S2 Fig.**). Indeed, mitomycin-c, a DNA crosslinker known to induce apoptosis in mammalian cells, showed a strong and significant increase in both caspase-3 activity in animals and Apotracker-Green in cells.

2. Reviewer comment: Regarding the timeline for the apoptotic response to treatment, it would be helpful to receive context from previous experiments (other studies) as to the typical temporal response after inoculation of Poly(I:C).

Response to reviewer:

Thank you for this comment. Indeed, we observed a similar pattern in the RNAseq data of our previously published paper by Lewandowska et.al (2021) (https://academic.oup.com/mbe/article/38/10/4546/6310749) in which the apoptosis related genes were maximally expressed at 24 hours and diminished within 48 hours (Figure 1 of our study). In agreement with this result, caspase-3 activity was the strongest within 24 hours. This is also mentioned in the text.

3. Reviewer comment: Why was the negative control for apotracker performed using a mock transfection but the colorimetric assay for cas-3 was performed with NaCl, or am I missing something? Please clarify.

Response to reviewer:

The mock transfection was used as a control for the *ex-vivo* transfection experiments measured by Apotracker green (see first comment), whereas the NaCl control was used for the *in-vivo* microinjection experiments measured by the caspase-3 colorimetric assay.

4. Reviewer comment: Regarding the phylogenetic analysis, in order to put the caspases into a larger phylogenetic context, I suggest to add annotations of well-studied representatives of other phylogenetic clades. This would greatly help follow the evolutionary signal (the annotation is currently a bit cumbersome).

Response to reviewer:

We agree with this comment and have accordingly updated Figure 5. A yellow star now marks *Homo sapiens*, *Mus musculus*, *Drosophila melanogaster* and *Caenorhabditis elegans*. Four well-studied species.

We added this to the figure legend:

*"**Fig 5**. Phylogenetic relationship among metazoan caspases made with the full-length sequence alignment generated by MAFFT. Numbers on nodes, separated by slashes, represent SH-aLRT, approximate Bayes test, and ultrafast bootstrap support values, in that order, for Maximum Likelihood (ML) analysis. Navajo's rug below the nodes illustrates the results of the sensitivity analysis (using different alignments) based on maximum likelihood. Grey squares means the clade is recovered in the analysis, white with an X ones were not. The domain organization is displayed alongside each clade. CASc: caspase domain, CARD: caspase-recruitment domain, DED: death-effector domain, NHR: neuralized homology repeat. Sequences from N. vectensis are highlighted in bold magenta and presented in a larger font. Some of these sequences feature an asterisk, indicating that these genes are upregulated at the 24hpi time point with a Log2 Fold Change > 1. A yellow star placed in front of certain branches denotes well-studied species (Cel: Caenorhabditis elegans, Dme:Drosophila melanogaster, Hsa: Homo sapiens and Mmu: Mus musculus). Rfi: Reticulomyxa filosa, Aqu: Amphimedon queenslandica, Adi: Acropora digitifera, NVE: N. vectensis vectensis, Sca: Scolanthus callimorphis, Ate: Actinia tenebrosa, Dme: Drosophila melanogaster, Dre: Danio rerio Hsa: Homo sapiens, Mmu: Mus musculus, Spu: Strongylocentrotus purpuratus, Cgi: Crassostrea gigas, Cte: Capitella teleta, Sko: Saccoglossus kowalevskii, Mvi: Morbakka virulenta, Aau: Aurelia aurita, Edi: Exaiptasia diaphana, Spi: Stylophora pistillata, Hvu: Hydra vulgaris, Che: Clytia hemisphaerica, Cel: Caenorhabditis elegans, Mle: Mnemiopsis leidyi, Bbe: Branchiostoma belcheri, Tsp: Trichoplax sp., Hsy: Hydractinia symbiolongicarpus".*

5. Reviewer comment: It is not totally convincing that the authors actually observe apoptosis and not another kind of immune-related cell death. The authors discuss this point shortly in the discussion, yet I find this not sufficient. The authors may consider analyzing their transcriptomics

data and genomics data for pyroptosis (or necroptosis) related genes. At least they should further discuss this possibility.

Response to reviewer:

We agree that this is an important suggestion and have included a new figure (**Fig 2**), which presents the findings from our analysis of the *N. vectensis* genome and our review of the reanalyzed gene expression data previously published by Lewandowska et al. (2021), which was used for this study. This analysis helped identifying the presence or absence of genes within the genome and their expression profiles in the transcriptome related to the necroptosis and pyroptosis pathways. We now added this section in the *Results:*

"Importantly, other types of programmed cell death, such as necroptosis and pyroptosis, have been reported in the context of the antiviral immune response in mammals [55-57]. Nonetheless, we did not identify any human homologous components of necroptosis in the N.vectensis genome and transcriptome (Fig 2). We did find a homolog (NVE20414) of Gasdermin E, a precursor of a pore-forming protein which mediates pyroptosis in mammals [58, 59], to be upregulated in our transcriptomic data (LFC=1.8, p=0.0014), suggesting that pyroptosis might also be activated. However, we could not identify homologs for Caspase-1 and NLRP3 in the N. vectensis genome, which are crucial for inducing pyroptosis in mammals [60, 61], in our genomic and transcriptomic analyses (Fig 2)".

And in the *Discussion:*

"In recent years, a growing body of evidence indicate that other forms of cell death are also involved in the antiviral response. In particular, necroptosis, a regulated form of necrotic cell death , and pyroptosis, a regulated form of inflammatory lytic cell death mediated by gasdermin proteins [56], are important host defense strategies for eliminating viral infected cells. The latter was observed in Cnidaria in the context of the antibacterial response [46, 47]. Our phylogenetic analysis revealed that the N. vectensis gene (NVE20429) orthologous to the Hydra vulgaris gene (Fig 5) which was recently reported to be involved in pyroptosis was not upregulated upon poly(I:C) treatment. However, a Gasdermin-encoding gene (NVE20414) was upregulated (LFC=1.8, p=0.0014) at 24 hpi. Importantly, gasdermins are regulated post-translationally by proteolytic cleavage [116]. Therefore, based on our data we cannot exclude the possibility that pyroptosis is also activated in response to dsRNA in N. vectensis (Fig 2). Further research is required to determine whether pyroptosis and apoptosis are activated simultaneously or whether these two processes are mutually exclusive in N. vectensis".

6. Reviewer comment: Relating to the previous point, the manuscript could be strengthened if the actual morphological phenotype was observed (by microscopy), diminishing the possibility of other mechanisms of cell death being at play which is followed by the apotracker expeirments.

Response to reviewer:

Thank you for this constructive comment. We fully agree. We now included two lines of experimental evidence indicative of apoptosis as the main form of programmed cell death in the context of dsRNA challenge:

1) The fraction of cells that were Apotracker Green$^-$ SYTOX Blue$^+$, which contains cells that have died due to reasons other than apoptosis, did not change significantly upon poly(I:C) transfection (p=0.445487, n=3, two-sided t-test) (**Fig. 3E**).

2) Imaging cytometry examination of cells from animals injected with poly(I:C) showed morphological alterations characteristic of apoptosis. These changes, including membrane

blebbing, the formation of apoptotic bodies, and cell shrinkage, were identified using Apotracker Green staining combined with the viability dye Zombie NIR (**S3 Fig.**).

Despite this evidence, it is still possible that other forms of cell death also play a role in this context. In fact, pyroptosis and necroptosis were described in mammals in the context of the antiviral response (see: https://www.cell.com/trends/microbiology/abstract/S0966-842X(21)00277-8, and: https://www.nature.com/articles/s41418-018-0172-x). We discussed these possibilities in the *discussion* and clarify that: "Based on our data we cannot exclude the possibility that pyroptosis is also activated in response to dsRNA.".

7. Reviewer comment: I believe that in terms of organization, the manuscript might have been more accessible and seamless if the results would begin with the transcriptomics analysis of the previous paper

Response to reviewer:

We fully agree with this comment. We now changed the order of the *Results* section and it now starts with the transcriptomic analysis.

Reviewer 2

Reviewer comment: In the results, please describe in one sentence the procedure of identifying Caspase homologs in Cnidarians (prior to diving into the phylogenetic analysis). In particular, in which clade did the authors search for Caspase homologs? How many hits were obtained in total? This information could be provided in one or two sentences. Then, in the methods, please provide more information about the database in which you searched for homologs of human caspases : how many genomes? how many hits on average per genome within each taxon ?

Response to reviewer: Thank you for the recommendation. We agree and this has been added to text.

"To broaden our understanding of the evolutionary history of caspases in Metazoans, we conducted tBLASTn and BLASTp searches of caspases on NCBI, Uniprot, and on available genomes of Metazoa representing Ctenophora, Porifera, Cnidaria, Protostomia, and Deuterostomia. We idetenfied 160 putative caspases and of these six putative caspase genes were identified in the genome of N. vectensis (S2 File)".

"Putative metazoan caspases were discovered using tBLASTn and BLASTp searches with eleven human caspases sourced from Uniprot as initial queries. These queries were then used on NCBI, Uniprot, and downloaded genomes. Subsequently, reciprocal BLAST was conducted, and the query was expanded based on the obtained results. Sequences with an e-value inferior to 1e-5 were retained. We conducted BLAST searches on the genomes of various species, including one Ctenophora, one sponge, nine cnidarians (five anthozoans and four medusozoans), two protostomes and two deuterostomes. All identified sequences were analyzed with SMART [128] and InterProScan (EMBL-EBI) to verify the presence of caspase (CASc) domains. A full list of all protein sequences used in the BLAST search and phylogenetic analysis are provided in supplementary file 2. On average, we identified six homologs in the genome of each species. Reticulomyxa filosa (Foraminifiera) caspase homologues were used as outgroup. Protein sequence alignments were generated for full-length sequence (all domains) and only for caspase domain (Casc) using the L-INS-i algorithm in MAFFT software version 7 [129], with default parameters".

Reviewer comment: Please add a supplementary figure being a phylogenetic tree of all animals (or all Eumetazoa) showing in which taxa each Caspase is present (e.g. using one outer ring/colour per known Caspase). This would really help the reader to understand which Caspase is present in which taxon (e.g. to understand very easily that Casp9 appeared only later in animal evolution.

Response to reviewer: Thank you for the recommendation. The supplementary figure has been added (**S8 Fig.**) and cited properly in the main text.

"Utilizing these phylogenetic results along with insights gleaned from the literature [88], we reconstructed the distribution of various caspase types across Metazoa (S8 Fig). The evolutionary history of caspases in Metazoa is particularly noteworthy, as all metazoan groups share one specific type: executioner caspases. Additionally, the frequent losses observed in the phylum Nematoda highlight a complex evolutionary trajectory for this protein family, characterized by numerous losses, acquisitions, and diversifications".

<u>Reviewer comment:</u> How did the authors define Clades I to VI in the phylogenetic tree? According to UFBoostrap values, some clades are not very well supported (when UFBoostrap < 80, the authors should consider that a clade is not well supported at all whereas only clades with UFBoostrap > 95 should be considered as well supported).

<u>Response to reviewer:</u> It is true, the interpretation of ultrafast bootstrap (UFBoot) tells us that one should probably believe in a clade if its support is >= 95%. Hence, we agree with this comment. In the previous version, we had placed this paragraph to specifically address this problem only for clade IV: "*This clade exhibits low bootstrap support, yet high SH-aLRT and aBayes support. This discrepancy could arise from a matter of statistical sensitivity, wherein SH-aLRT and aBayes tests may be more sensitive to certain data patterns. They might detect signals in the data supporting the branch that bootstrap analysis might miss, especially if the dataset is small or the signal is weak [73]."* In the revised version of the manuscript, we have moved the paragraph and made modifications to it. It includes the results of the additional analyses you recommended (see the following comment), providing more comprehensive evidence supporting the definition of the clades.

"*Although the ultrafast bootstrap values supporting the six delineated clades (I-VI) exceed 95 only for clades I and II, the support for the remaining four clades ranges from 16 to 81. Notably, clade IV is the only one with a bootstrap value below 70. Despite this, we obtain high support from both the SH-aLRT and the Bayes test for these clades (**Fig 5**). This discrepancy could arise from a matter of statistical sensitivity, wherein SH-aLRT and aBayes tests may be more sensitive to certain data patterns. They might detect signals in the data supporting the branch that bootstrap analysis might miss, especially if the dataset is small or the signal is weak [87]. Furthermore, it is important to note that the results of the five phylogenetic analyses, including two phylogenetic analyses based on only the Casc domains, and three analyses based on full length sequence were highly congruent regarding the tree structure and particularly in identifying the six clades delineated here (I-VI), demonstrating the robustness of the results (**Fig 5** and **S7A-D**). This is illustrated in figure 5 using navajo rugs representing the results of the sensitivity analysis."*

In the following comment, we detail all text modifications and additions which are related to this issue.

<u>Reviewer comment:</u> Was the Multiple Sequence Alignment built on the full length of each protein? We would ask the authors to also compute a Multiple Sequence Alignment only on the Caspase (CASc) domain. This would first allow to get a better MSA (when looking at the one the authors provided, it seems that best aligned region is really the one corresponding to this domain vs. the N-Terminal part of proteins). With a better alignment, one also generally gets better support values for the different clades which would make the clade I-IV definition more convincing. Finally, it is always interesting to compare trees obtained at the protein level vs. at the domain level. Optionally, the authors could also use alternative aligners such as Muscle (with the super5 algorithm) in order to test if the tree topology is robust to this type of change. The authors could provide in supplementary figure the trees obtained using this aligner in order to show the robustness of their results.

section: Methods

"**Sequence dataset construction**

Putative metazoan caspases were discovered using tBLASTn and BLASTp searches with eleven human caspases sourced from Uniprot as initial queries. These queries were then used on NCBI, Uniprot, and

downloaded genomes. Subsequently, reciprocal BLAST was conducted, and the query was expanded based on the obtained results. Sequences with an e-value inferior to 1e-5 were retained. We conducted BLAST searches on the genomes of various species, including one Ctenophora, one sponge, nine cnidarians (five anthozoans and four medusozoans), two protostomes and two deuterostomes. All identified sequences were analyzed with SMART [126] and InterProScan (EMBL-EBI) to verify the presence of caspase (CASc) domains. A full list of all protein sequences used in the BLAST search and phylogenetic analysis are provided in supplementary file 2. On average, we identified six homologs in the genome of each species. Reticulomyxa filosa (Foraminifiera) caspase homologues were used as outgroup. Protein sequence alignments were generated for full-length sequence (all domains) and only for caspase domain (Casc) using the L-INS-i algorithm in MAFFT software version 7 [127], with default parameters. In addition, we generated multiple alignments using the MUSCLE software [128] with default parameters, both for the full sequence and only for the caspase domain (Casc). Four final multiple alignments were generated to assess the topological congruence across different alignments".

"**Tree reconstruction**

*Phylogenetic analyses for the four multiple alignments previously generated were conducted using amino-acid alignment (**S4 File**), employing the Maximum-Likelihood (ML) method in IQ-TREE2 [129] and Bayesian Inference (BI) analysis conducted in MrBayes version 3.2.6 [130], respectively. Models of protein sequence evolution for ML (AIC criteria) and BI (BIC criteria) analyses were estimated using ModelFinder [131]. Branches support of the ML tree was assessed through 1000 ultrafast bootstrapping replicates (UBS) [132], 1000 bootstrap replicates for SH-aLRT [87] and an approximate aBayes test [133]. For the bayesian inference analysis, we used only the final multiple alignment of the full sequence, which was generated by MAFFT. Two independent runs were carried out, each with four simultaneous Markov chains over 15,000,000 generations and sampled every 1000 generations. Convergence was checked using Tracer [134]. The consensus tree, along with the posterior probabilities of clades, was derived from trees sampled across the two runs. 25% of the sampled trees were discarded as burn-in. The resulting trees were visualized using figtree version 1.4.4 (http://tree.bio.ed.ac.uk/software/figtree/)".*

Section: Results

"*Five phylogenetic analyses were conducted to explore the distribution of homologs of these caspase genes from N. vectensis across Metazoa. The Maximum-Likelihood tree depicted in **Fig 5** was inferred from full-length sequence alignment generated by MAFFT. Although the support values are very similar across all analyses, this result displays the highest support values. This phylogenetic tree shows that ..."*

"Furthermore, it is important to note that the results of the five phylogenetic analyses, including two phylogenetic analyses based on only the Casc domains, and three analyses based on full length sequence were highly congruent regarding the tree structure and particularly in identifying the six clades delineated here (I-VI), demonstrating the robustness of the results (**Fig 5** and **S7A-D**). This is illustrated in figure 5 using navajo rugs representing the results of the sensitivity analysis."

Figure legend:

Fig 5. *Phylogenetic relationship among metazoan caspases made with the full-length sequence alignment generated by MAFFT. Numbers on nodes, separated by slashes, represent SH-aLRT, approximate Bayes test, and ultrafast bootstrap support values, in that order, for Maximum Likelihood (ML) analysis. Navajo's rug below the nodes illustrates the results of the sensitivity analysis (using different alignments) based on maximum likelihood. Grey squares means the clade is recovered in the analysis, white with an X ones were not. The domain organization is displayed alongside each clade. CASc: caspase domain, CARD: caspase-recruitment domain, DED: death-effector domain, NHR: neuralized homology repeat. Sequences from N. vectensis are highlighted in bold magenta and presented*

in a larger font. Some of these sequences feature an asterisk, indicating that these genes are upregulated at the 24hpi time point with a Log2 Fold Change > 1. A yellow star placed in front of certain branches denotes well-studied species (Cel: Caenorhabditis elegans, Dme:Drosophila melanogaster, Hsa: Homo sapiens and Mmu: Mus musculus). Rfi: Reticulomyxa filosa, Aqu: Amphimedon queenslandica, Adi: Acropora digitifera, NVE: N. vectensis vectensis, Sca: Scolanthus callimorphis, Ate: Actinia tenebrosa, Dme: Drosophila melanogaster, Dre: Danio rerio Hsa: Homo sapiens, Mmu: Mus musculus, Spu: Strongylocentrotus purpuratus, Cgi: Crassostrea gigas, Cte: Capitella teleta, Sko: Saccoglossus kowalevskii, Mvi: Morbakka virulenta, Aau: Aurelia aurita, Edi: Exaiptasia diaphana, Spi: Stylophora pistillata, Hvu: Hydra vulgaris, Che: Clytia hemisphaerica, Cel: Caenorhabditis elegans, Mle: Mnemiopsis leidyi, Bbe: Branchiostoma belcheri, Tsp: Trichoplax sp., Hsy: Hydractinia symbiolongicarpus.

Reviewer #3: No Major Issues are present in the current study.

Part III – Minor Issues: Editorial and Data Presentation Modifications
Please use this section for editorial suggestions as well as relatively minor modifications of existing data that would enhance clarity.

Reviewer 1

Reviewer comment:

- Lines 243 – 252 belongs in the discussion

Response to reviewer:

We agree with this comment and we now moved this part to the *Discussion* section.

Reviewer comment:

- Organization of figure 2: for comparison a,b,c should be in one line

Response to reviewer:

We agree with this comment, and we moved the panels accordingly.

Reviewer 2

Reviewer comment: Figure 1 could be smaller (e.g. panels B and D could be reduced).

Response to reviewer: We modified this figure (now Figure 3) and it now includes more information (7 homogenized items) on a similar space.

Reviewer comment: What are the three pairs of curves representing in Figures 1A and 1C ? We suppose this is three time points (6h, 24h and 48h) but the information is not provided in the Figure description. The authors should provide this information.

Response to reviewer: Thank you for this comment. We modified this figure (now Figure 3) and it now includes a representative replicate of each condition with labelled axes. We think the figure is now easier to understand and still as informative as the original since the statistics is shown as a bar plot.

Reviewer comment: The same y-axis should not be used for the three pairs of curves in Figures 1A and 1C and ticks should be added (even though this is only 0 and 1 for normalized

Response to reviewer: Thank you for this comment. We modified this figure (**Fig. 3**) and it now includes a representative replicate of each condition with labelled axes and ticks.

Reviewer comment: Page 7 "whereas caspase activity […] did not reach a statistical significance at 48hpi" : what do the authors conclude from that ? Do they expect that the response should decrease after 48h ?

Response to reviewer: Based on the transcriptomic analysis (**Fig. 1**), indeed, we expected the strongest response to be within 24 hours. In agreement with the transcriptomic analysis (**Fig. 1A and 1B**), the response to poly(I:C) was strongest at 24 hpi. As expected, the response was not detectable within 6 hpi and decreased within 48 hours. The paragraph now reads:

*"**...** Next**,** we compared the Caspase-3 activity in poly(I:C) injected animals versus NaCl control at different time points. Three batches of zygotes were used as biological replicates for each condition. At 6 hpi, we did not observe a significant difference in Caspase-3 activity compared to NaCl control (p=0.56, n=3, one-sided t-test) (**Fig 4A**), whereas caspase activity was significantly increased at 24 hpi (p=0.0013, n=3, one-sided t-test) and did not reach a statistical significance at 48 hpi (p=0.069,n=3, one-sided t-test) (**Fig 4B and 4C**). The increase in Caspase-3 activity relative to 6 hours was most noticeable at 24 hours (p=0.145, n=3, one way ANOVA test) after injection and decreased in magnitude at 48 hpi (p=0.469, n=3, one way ANOVA test), however, these differences did not reach statistical significance (**Fig 4D**). In agreement with our transcriptomic analysis (**Fig. 1A and 1B**), the response to poly(I:C) was strongest at 24hpi. Altogether, these results indicate that poly(I:C) is sufficient to activate Caspase-3 in vivo in a time dependent manner."*

Reviewer comment: Page 7 "which is not orthologous of TLR3" : Missing reference.

Response to reviewer: Thank you for catching this error. We now added the reference.

Reviewer comment: Paragraph "dsRNA induces apoptosis in N. vectensis injected animals" : it feels like this paragraph lacks an introduction and a conclusion to really link with what comes before and after. What does the paragraph brings to the whole ? I think stating it clearly will help the reader to follow the story.

Response to reviewer: Thank you for this comment. We modified this paragraph to emphasize the importance of this assay for studying apoptosis *in vivo*. It now reads:

"*Caspases are key mediators of apoptosis. Among them, Caspase-3 is a major executioner caspase involved in the execution phase of apoptosis [75]. Following cleavage and activation by initiator caspases (CASP8, CASP9 and/or CASP10), the mammalian Caspase-3 executes apoptosis by catalyzing cleavage of many essential key proteins [75-78]. Methods for determining Caspase-3 activity are based on the proteolytic cleavage of its preferred recognition sequence Asp-Glu-Val-Asp (DEVD) [79]. Such methods have been applied for studying apoptosis upon stress conditions in other cnidarians including corals and the sea anemone Exaiptasia diaphana [11, 80, 81]. To study the effect of dsRNA in vivo we first validated this assay by incubating zygotes with 60 µM MMC and compared them to DMSO treated controls. Indeed, 24 hours MMC treatment resulted in about 2-fold increase in Caspase-3 activity (**S6A Fig**), whereas 48 hours incubation resulted in a slightly stronger activity of about 2.75-fold increase (**S6B Fig).** Next**,** we compared the Caspase-3 activity in poly(I:C) injected animals versus NaCl control at different time points. Three batches of zygotes were used as biological replicates for each condition. At 6 hpi, we did not observe a significant difference in Caspase-3 activity compared to NaCl control (p=0.56, n=3, one-*

*sided t-test) (**Fig 4A**), whereas caspase activity was significantly increased at 24 hpi (p=0.0013, n=3, one-sided t-test) and did not reach a statistical significance at 48 hpi (p=0.069,n=3, one-sided t-test) (**Fig 4B and 4C**). The increase in Caspase-3 activity relative to 6 hours was most noticeable at 24 hours (p=0.145, n=3, one way ANOVA test) after injection and decreased in magnitude at 48 hpi (p=0.469, n=3, one way ANOVA test), however, these differences did not reach statistical significance (**Fig 4D**). In agreement with our transcriptomic analysis (**Fig. 1A and 1B**), the response to poly(I:C) was strongest at 24hpi. Altogether, these results indicate that poly(I:C) is sufficient to activate Caspase-3 in vivo in a time dependent manner*."

Reviewer comment: Figure 3 : The analyzed dataset comes from Lewandowska et al., right ? Please specify it in the Figure legend.

Response to reviewer: We have added this information in the legend of Figure 1 legend (Figure 3 is now Figure 1).

Reviewer comment: Figure 3A : It is unclear to us why some genes appear several times in the volcano plot (e.g. Caspase 3/6/7 or Bcl2-like). Please precise this in the Figure legend.

Response to reviewer: Thank you for catching this error. This has been fixed; Figure 3 is now figure 1. We have added a tab (1. Genes) in the supplementary file 1, where the equivalence of the *Nematostella vectensis* gene models can be found (upregulated genes showed in the figure 1A) and their corresponding gene names.

Reviewer comment: In Figure 4, the authors could indicate which prefix corresponds to which species with some taxonomic information (e.g. edi = Exaiptasia diaphana) in order to guide the reader.

Response to reviewer: Thanks for the suggestion. We had added this to the Figure 5 legend.

Reviewer comment: Page 12 : "dsRNA induces a conserved antiviral response pathway across metazoans" : isn't it Eumetazoans instead ? Metazoans is more broad (it includes e.g. Porifera).

Response to reviewer: We included the Porifera phylum in our study because it represents one of the phylogenetically oldest group of metazoans, and it is predicted to have an apoptotic machinery. For the phylogenetic reconstruction, we utilized homologs derived from this group (sponges).

Reviewer 3

General comments:

Reviewer comment:
The references of all products used in this study need to be added and homogenized in the entire manuscript. All figure names need to be corrected in regard to the name of the figure in the figure legends and homogenized in the entire manuscript.
Response to reviewer: Thank you for noticing this problem. We fixed this issue in the revised manuscript.

Reviewer comment:

P3 Introduction
L102 - Nevertheless – Typo

Response to reviewer: We replaced Nevertheless with However.

Reviewer comment:

L111 – Syntax:
Replace: “In this study” by “In the current study” as the first form seems to refer to the previous study performed by the authors (ref 52).
Response to reviewer: Done.

Reviewer comment:
L113-114: “and uncovered a conserved network of genes involved in this process”,
Complete the sentence. Conserved to what? If there is one, what is the degree of conservation?
Response to reviewer: Thank you for this comment. We rephrased this sentence and it now reads:"We found that the dsRNA viral mimic poly(I:C) is sufficient to strongly induce apoptosis in *N. vectensis* and uncovered a gene network involved in this process in *N. vectensis* and various bilaterian groups, pointing to a functional conservation of dsRNA-induced apoptosis that existed before the cnidarian-bilaterian split."

Reviewer comment:
P6: dsRNA induces apoptosis in N. vectensis derived cells ex-vivo
L121-122:” We first used mitomycin-c (MMC) and cycloheximide (CHX) treatments… as positive controls to validate this assay in N. vectensis”
Q: - Do you mean AND = both treatment at the same time or do you mean OR independent treatment? clarify.

Response to reviewer: We used these two positive controls independently as highlighted now in the revised manuscript. It now reads: “We first used either mitomycin-c (MMC) or cycloheximide (CHX) treatments, common inducers of apoptosis in human and mouse cells [1, 2], as two independent positive controls to validate this assay in *N. vectensis*.”

Reviewer comment:
Q: - As there is lots of autofluorescence in the green and red in Nematostella tissues/cells, how do you make the difference between the autofluorescence of the tissues/cells and the specificity of your staining Apotracker-green (Apo-15)?

Response to reviewer: We now added a histogram showing the SYTOX Blue single stain in S4A Fig. As shown, the green autofluorescence (noise) is negligible (shown on a logarithmic scale) relative to the Apotracker Green signal.

Reviewer comment:
L127 :” resulted in a significantly increased Apotracker green fluorescence intensity compared to DMSO”
Q: - What do you mean by significant? Could you precise the values in the text please.

Response to reviewer: We provide the p-value: “Treatment of *N. vectensis* derived cells with 2 mM of the translation blocker cycloheximide for 48 hours resulted in a significantly increased Apotracker Green median fluorescence intensity (MFI) compared to DMSO (p=0.001269, n=3, two-sided t-test) (**S1A and S1B Fig**).”

Reviewer comment:
Q: - How can you explain to have such high numbers of Apotracker green positive cells in your DMSO control?

Response to reviewer: That is a good point. DMSO at high concentrations can be toxic and can induce apoptosis by itself. This phenomenon is well-documented for mammalian cells. Please refer to this paper as an example: https://faseb.onlinelibrary.wiley.com/doi/abs/10.1096/fj.13-235440

Reviewer comment:
L128 :
Typo/replace : “Supplementary figure 1B, 2A, 2B” by “Supplementary figure 1b, 2a, 2b”.

Response to reviewer: We corrected this problem.

Reviewer comment:
L131:
Typo/replace: “Supplementary figure 1C” by “Supplementary figure 1c”.
Please homogenize the number in all the manuscript to stay consistent.

Response to reviewer: We corrected this problem and homogenized the figure numbers throughout the text according to the journal style.

Reviewer comment:
L132:
Complete this sentence ”was previously used to kill proliferating cells before stem cell transplantation in N. vectensis” by ” was previously used to kill proliferating cells before candidate stem cell transplantation in N. vectensis”. In fact the original pre-print uses the term candidate stem cells as the definite evaluation of being stem cells remains to be shown.

Response to reviewer: We agree with this remark. It now reads the same as the reviewer suggested.

Reviewer comment:
L134:
Typo: replace : “Supplementary figure 2C, 2D” by “Supplementary figure 2c, 2d”.

Response to reviewer: We corrected the name of the figures according to the PLOS Pathogens format throughout the entire manuscript.

Reviewer comment:
L143:
”To deliver poly(I:C) into cells, we transfected N. vectensis-derived cells with 2 µg/ml poly(I:C).”
Q: - why did you choose this concentration? Please justify.

Response to reviewer: This concentration is commonly used in mammalian cells. We now added two references that used concentrations within the range of 0.4-10 µg/ml in human cell lines. The text now reads: “To deliver poly(I:C) into cells, we transfected *N. vectensis*-derived cells with 2 µg/ml poly(I:C), a concentration that is commonly used for transfecting human cell lines [70, 71]”.

Reviewer comment:
L144-145:” we detected a significantly increased Apotracker green fluorescence intensity in poly(I:C) transfected cells relative to their mock transfected counterparts”
Complete this sentence using datas/numbers to support the significance.

Complete Figure 1a, there are no units on the vertical axis.
Clarify what are the six curves shown in this graph.

Response to reviewer: We provide the p-value: "We detected a significantly increased Apotracker Green MFI in poly(I:C) transfected cells relative to their mock transfected counterparts (p=0.0265, n=3, one-sided t-test) (**Fig 2A-C**). Regarding the figure, we now show a representative dot plot with labelled axes and provide the statistics as a bar plot.

Reviewer comment:
L145 :
Typo: replace : "(Figure 1A, 1B)" by "(Figure 1a, 1b)"
Please homogenize throughout the text.

Response to reviewer: Thank you for commenting on this issue. We now corrected it throughout the text (following the journal style).

Reviewer comment:
L150:"
about 29 % to 44 % (Supplementary figure 3A-C)."
Q: - what is the total number of the cells?

Response to reviewer: The total number of recorded events was 30,000 as mentioned in the figure legend: "30,000 events were acquired for each replicate. 3 biological replicates were performed for each condition".

Reviewer comment:
P7: dsRNA induces apoptosis in N. vectensis injected animals
L164 :" injected animals versus NaCl control"
versus in italic.

Response to reviewer: Versus now appears in italic.

Reviewer comment:
Figure 2a, b and c. n =3 :
Q: - Do you mean that these experiments include only "3 zygotes" or "3 batches of zygotes"? Clarify.

Response to reviewer: Thank you for this comment. We mean 3 batches of zygotes and we now incorporated this clarification in the text. The figure legend now reads: "Three biological replicates each of which consisting of 3 batches of zygotes were used for each condition." And in the main text as follows: "Next**,** we compared the Caspase-3 activity in poly(I:C) injected animals *versus* NaCl control at different time points. Three batches of zygotes were used as biological replicates for each condition".

Reviewer comment:
P8: dsRNA triggers the activation of genes associated with the apoptotic pathway
L174: " Lewandowska et al. (2021)"
Add in the text that this corresponds the reference (52) in your bibliography.

Response to reviewer: The sentence now reads:

"Raw reads files obtained from Lewandowska et al. 2021 [52] were aligned to N. vectensis reference genome (NCBI accession number: GCA_000209225.1) using STAR version 2.7.10a [117]"

Reviewer comment:
L176-178: "expression pattern strongly associated with apoptotic process including upregulated expression of genes encoding initiator and executioner caspases (Caspase-8 and Caspase-3/6/7), negative apoptotic regulators (Bcl, MCL and IAP), and positive apoptotic regulators (Bok, Apaf-1 and TRAFs) for 24 hpi."
In order to clarify and complete this point, please add in the text the number of fold change that supports this idea for each gene of interest listed in the text.

Response to reviewer: We have added the values of log_2 fold change for each gene.

"..... of genes encoding initiator and executioner caspases (Caspase-8 and Caspase-3/6/7-like1, with Log_2 fold change (LFC) of 2.84 and 3.37, respectively), negative apoptotic regulators (Bcl-2-like3, MCL and IAP, with LFC of 1.39, 2.86 and 1.4, respectively), and positive apoptotic regulators (Bok, Apaf-1 and TRAF3-like1, with LFC of 3.63, 1.12 and 1.85) at 24 hours post injection (hpi)."

Reviewer comment:
L181: "No upregulated apoptosis-related genes were detected at 6 hpi (Figure 3A, Supplementary file 1)."
This idea is not supported by any data in the current paper, maybe a figure is missing, could you complete/clarify please.

Response to reviewer: Thank you. We have addressed this by modifying the sentence for clarity and adding data supporting the idea.

"34 upregulated genes were detected at 6 hpi and none of them were related to apoptosis (S1 File: Table 2)."

Reviewer comment:
L184-185:" Furthermore, based on the identified upregulated genes associated with apoptosis in N. vectensis at 24 hpi, it seems that both the intrinsic and the extrinsic pathways of apoptosis were activated"
Please support your idea showing the data ex. by listing them in the text or in an identified separate table, highlighting the upregulated genes of interest for the intrinsic versus extrinsic apoptotic pathway.

Response to reviewer: Thank you for this suggestion and the next one that are related. We added a sentence to clarify the connection between these two ideas or paragraphs.

*" ..... it seems that both the intrinsic and the extrinsic pathways of apoptosis were activated. Considering only the upregulated genes associated with apoptosis identified at 24 hpi, we propose and illustrate a hypothetical model for both the intrinsic and extrinsic pathways of apoptosis in N. vectensis. This model is based on homology and existing knowledge from the bilaterian system (**Fig 1C**). The intrinsic pathway (**Fig 1C**) ....."*

Reviewer comment:
L185-197:" The intrinsic pathway (depicted on the left in Figure 3C) may be initiated by various intracellular signals, such as DNA damage. These signals lead to the downregulation of pro-survival proteins like Bcl-2 and Bcl-10, thereby enabling pro-apoptotic molecules such as Bok, to provoke a mitochondrial permeability transition. This transition results in the release of cytochrome c and other pro-apoptotic molecules, counteracting the anti-apoptotic activity of IAPs. Following this, cytochrome c

associates with Apaf-1 to form the apoptosome, which activates initiator caspases. Subsequently, initiator caspases trigger the executioner caspase cascade, culminating in cell death. The extrinsic pathway (Figure 3C) is initiated by the binding of secreted ligands from the TNF family to their corresponding receptors within the TNFR family. Upon activation, receptor homotrimers or homomers recruit cytoplasmic adaptor molecules, which then instigate cell death through various pathways. Following the activation of classical cell death receptors (e.g., FAS, DR4/5, TNFR1), adaptor molecules directly bind and facilitate the cleavage of initiator caspases. These, in turn, activate the effector caspases that orchestrate cell death [68]."

This paragraph of the result section only contains data from other papers. I see two options here: Either this paragraph as it, is included in the discussion or if left in the current section data from the current RNAseq need to be added.

Response to reviewer: Thank you. We retained the paragraph in the results section and followed the Reviewer's suggestion by incorporating information from the RNA-seq analysis. We think that with these two modifications the objective here is now clearer and the idea supported.

*" The intrinsic pathway (**Fig 1C**) may be initiated by various intracellular signals, such as DNA damage. These signals lead to the downregulation of pro-survival proteins like Bcl-2 and Bcl-10, thereby enabling pro-apoptotic molecules such as Bok (At 24 hpi, a homolog was found to be upregulated in N. vectensis, with a LFC value of 3.63), to provoke a mitochondrial permeability transition. This transition results in the release of cytochrome c and other pro-apoptotic molecules, counteracting the anti-apoptotic activity of IAPs [53]. Following this, cytochrome c associates with Apaf-1 to form the apoptosome (At 24 hpi, a homolog was found to be upregulated in N.vectensis, with a LFC value of 1.12), which activates initiator caspases (At 24 hpi, one initiator caspase was found to be upregulated in N. vectensis. Caspase-8 with a LFC value of 2.8). Subsequently, initiator caspases trigger the executioner caspase cascade, culminating in cell death (At 24 hpi, two homologs of executioner caspases were found to be upregulated. Caspase 3/6/7-like1 and Caspase 3/6/7-like2, with LFC values of 3.37 and 1.15 respectively). In contrast to the intrinsic pathway, the extrinsic pathway (**Fig 1C**) is initiated by the binding of secreted ligands from the TNF family to their corresponding receptors within the TNFR family (At 24 hpi, homologs of TNF receptor-associated factor were found to be upregulated. TRAF3-like1 and TRAF3-like2, with LFC values of 1.85 and 2.62 respectively). The sole distinction between the two pathways is that the extrinsic pathway involves death receptors at the initiation of the apoptotic cascade [53]. Upon activation, receptor homotrimers or homomers recruit cytoplasmic adaptor molecules, which then instigate cell death through various pathways. Following the activation of classical cell death receptors (e.g., FAS, DR4/5, TNFR1), adaptor molecules directly bind and facilitate the cleavage of initiator caspases. These, in turn, activate the executioner caspases that orchestrate cell death [54]**".***

Reviewer comment:

Caspases in Metazoa: Phylogenetic analysis and domain organization

While this section is very interesting, it needs to be complete with a phylogenetic analysis of the other apoptotic gene family members highlighted in this current study. In addition, this section needs to be completed by including and comparing the current data with a previous study looking at the apoptosis complement in cnidaria (Moya et al, 2016).

Response to reviewer: Thank you for this comment. Two phylogenies, corresponding to protein families involved in the apoptotic pathway (Apaf-1 and Bcl-2), were reconstructed. The resulting topologies are

presented in Supplementary Files 8A and 8B. Additionally, we have revised the text to include detailed information related to these two phylogenies.

Section: Methods

"… In addition to the caspase family, we also conducted a phylogenetic reconstruction of two protein families belonging to the apoptotic pathway that were found to be upregulated in our data: apoptotic protease activating factor-1 (Apaf-1) and B-cell lymphoma 2 (Bcl-2). A full list of all protein sequences used for the phylogenetic analysis is provided in supplementary file 2. Protein sequence alignments for these two protein families were generated for full-length sequence (all domains) using the L-INS-i algorithm in MAFFT software version 7 [127]".

" Phylogenetic analyses for the two protein families (Apaf-1 and Bcl-2) were conducted using amino-acid alignment, employing the Maximum-Likelihood (ML) method in IQ-TREE2".

Section: Results

"Bcl-2 and Apaf-1 protein sequences were retrieved from Uniprot, NCBI, and downloaded genomes. This was conducted using the methodology described above for the caspase family. The two topologies obtained are showed (S9A and S9B Fig). Nine putative Bcl-2 proteins were identified in N. vectensis and were dispersed throughout the phylogeny (S9A Fig). Five of these proteins clustered with representatives from the anti-apoptotic functional group, where two distinct groups, Bcl-2 and Bcl-b, were clearly identified. Additionally, three putative Bcl-2 proteins of N. vectensis clustered within the pro-apoptotic clades, Bax and Bok. Three putative Apaf-1 proteins were identified in N. vectensis (S9B Fig). NVE2160, the gene upregulated in our study, is clustered in a clade characterized by sequences that possess the CARD and NB-ARC domains, receiving the highest support. These results are similar to the phylogenies previously obtained in other studies for these two protein families [88, 89].

"

Reviewer comment:
L229: "is characterized by the sole presence of the CASc domain"
Add data/values to support your idea with data in a corresponding figure.

Response to reviewer: We have included the support values for this clade in the text, demonstrating the robustness of the inferred group.

Reviewer comment:
L242-243: "… a study that encompasses a comprehensive phylogenetic analysis of the caspase family was published [75]."
Q: - What about Moya et al, 2016: Functional conservation of the apoptotic machinery from coral to man: the diverse and complex Bcl-2 and caspase repertoires of Acropora millepora. In this paper the authors are highlighting several apoptotic genes from Nematostella including Caspases in their phylogenetic analysis.
https://openresearchrepository.anu.edu.au/bitstream/1885/95497/3/01_Moya_Functional_Conservation_2016.pdf
Please place these data regarding your data analysis in this result section.

Response to reviewer: Thank you for the suggestion. We have added this text to the *Discussion*.

"*Contrasting with Krasovec's et al. findings [88], the 2016 study by Moya et al. [89], which features a comprehensive phylogenetic analysis of the caspase family, is highly congruent with our conclusions. For instance, their analysis places the NEMV 201 sequence, equivalent to our NVE9681_casp8 sequence, closer to the clade they identify as 'Vertebrate caspase-8-10'. Similarly, the NEMV205 sequence, corresponding to our NVE26090_casp1/2/4/5/14 sequence, is also situated in the same clade, closely related to human caspase 2 and 14. This similarity may stem from Moya and collaborators' extensive sampling within the Cnidaria phylum, akin to the methodology of our study.*"

Reviewer comment:
L261-263: "in each study, we found that GO terms related to apoptosis are significantly enriched in all the studies, except for the hemichordate Saccoglossus kowalevskii, which did not show any significant GO terms related to apoptosis (Figure 5)."
Figure 5 does not show this specific analysis.
Complete the figure 5 with the data or correct the sentence.

Response to reviewer: Thank you for your suggestion. We amended some parts related to this analysis because the way it was written was confusing. With these modifications we want to emphasize that this analysis is a synthesis inferred from the results of other studies and therefore we refer to the tables, figures and supplements of those studies.

Section: Methods

"**Cross-species comparative transcriptomic analysis**

To contextualize our findings within a comparative evolutionary framework, we analyzed the transcriptomic response to dsRNA viral mimic poly(I:C) across seven species (*N. vectensis*, *Homo sapiens*, *Mus musculus*, *S. kowalevskii*, *Crassostrea gigas*, *Branchiostoma belcheri*, and *Schmidtea mediterranea*) from diverse metazoan phylogenetic lineages [52, 90, 91, 95, 113, 116, 135, 136]. Our analysis drew on results obtained from each corresponding study. The aim was to ascertain whether dsRNA impacts apoptosis by examining the outcomes of the gene ontology (GO) analysis conducted in each study, and consequently the outcomes of differential expression analysis conducted in each study, to identify if the genes within the apoptosis pathway are upregulated. Detailed information on each of the studies used is found in S3 File".

Section: Results

" dsRNA induces a conserved antiviral response pathway across metazoans

To obtain a wider picture on the evolution of the antiviral apoptotic response in animals, we analyzed the results from seven studies [52, 90-95] focused on the gene expression response to immune challenges (**Fig 6A**). Most of these studies specifically employed poly (I:C) as a viral mimic. Importantly, Bar Yaacov's 2022 study [90] on the planarian *Schmidtea mediterranea* employed a double-stranded RNA virus, rather than poly (I:C), as the immunological challenge. Despite differences in the species studied, experimental designs, and analytical methods, these studies collectively contribute to our understanding of dsRNA's influence on apoptosis on an evolutionary scale. Based on the results of the gene ontology (GO) analysis conducted in each study, we found that GO terms related to apoptosis are significantly enriched in all the studies, except for the hemichordate *Saccoglossus kowalevskii*, which did not show any significant GO terms related to apoptosis (**Fig 6B, S3 File**). Similarly, under the apoptosis categories, we pinpointed clusters of upregulated genes within crucial protein families in the apoptotic pathway, including caspases (both initiators and executioners), anti-apoptotic regulators like the Bcl-2 family members, and pro-apoptotic factors such as Apoptotic Protease-Activating Factor 1 (Apaf-1),

Tumor Necrosis Factor (TNFs), and TNF Receptor Associated Factor (TRAFs) (**Fig 6B**). The majority of lineages exhibited a shared upregulation of these genes."

Figure 6 legend:

"Fig 6. *Synthesis on the evolution of the antiviral apoptotic response in animals. (A) Flowchart depicting the steps to gather information on the studies of interest. (B) A simplified phylogenetic tree representing selected groups, highlighting the regulatory patterns of key apoptosis gene families. In this representation, a check mark (√) denotes the up-regulation of a gene, while an X indicates the absence of upregulation following exposure to the dsRNA viral mimic poly(I:C) or virus. *The study pertaining to Platyhelminthes utilized a dsRNA virus as opposed to poly(I:C). **This information is synthesized from two separate studies. The specific details of each study are available in supplementary file 3".*

Reviewer comment:
L267-268:" The majority of lineages exhibited a shared upregulation of these genes."
Data not shown. Provide the data supporting this idea or remove the sentence.

Response to reviewer: Thank you for this comment. This conclusion was extracted from the synthesis (**Fig. 6B**) and the results in each study were used as we explained in the previous comment.

Reviewer comment:
P12 Discussion
L291-292: "identified orthologs for all four caspase genes responsible for apoptosis in mammals, out of which 3 were upregulated upon poly(I:C) treatment."
Show this data or remove the sentence.

Response to reviewer: We amended this sentence. It now reads:

*" Our phylogenetic analysis (**Fig 5**) revealed* ***three orthologs that were upregulated following poly(I:C) treatment, corresponding to the two types of caspases (initiator and executioner) involved in mammalian apoptosis. NVE9681_casp8 belongs to clade III (Fig 5), which comprises exclusively initiator caspases, and was found to be upregulated at the 24 hpi time point with a LFC of 2.8, NVE23160_casp3/6/7 with a LFC of 1.15, and NVE21851_casp3/6/7 with a LFC of 3.37 at 24 hpi , both were identified in the clade VI (Fig 5), which comprises executioner caspases".***

Reviewer comment:

L293-294: "Our findings challenge the dichotomy found in the literature between the antiviral immune systems of vertebrates and invertebrates "
Q: - Which "dichotomy"? Complete the sentence.

Response to reviewer: We rephrased and completed the sentence and it now reads: "Our results challenge the established dichotomy in the literature regarding the antiviral immune systems of vertebrates, which predominantly depend on the interferon pathway, and invertebrates, which are believed to primarily rely on RNAi. It appears that *N. vectensis* can activate apoptosis similarly to vertebrates in response to dsRNA challenge".

Reviewer comment:
L297-299: "(1) most representative metazoans (6 out 7 analyzed) respond to dsRNA by significantly upregulating genes related to apoptosis, and (2) the upregulated genes were mostly conserved between distant species."

This idea needs to be clearly supported in the main core of the manuscript by adding a figure showing the entire analysis related to this conclusion.

Response to reviewer: Thank you for this suggestion. We added a flowchart at Figure 6 (now panel A) where we explain the entire analysis. This should be helpful for understanding this issue. Figure 6 was divided in two parts (A and B), where A is the flowchart and B is the synthesis.

Reviewer comment:
P15 M&M Primary cell culture:
L341:" Cell suspension with viability of more than 80 % was used for cell culture."
These data need to be presented and included in the paper.

Response to reviewer: We now added the range of viability used in our experiments:

"*Cell suspension with viability of more than 80 % (ranging from 81.7% to 91.1%) was used for cell culture."*.
